# Pyoverdine Plays Only a Minor, Strain‐Specific Role in the Inhibition of *Phytophthora infestans* by *Pseudomonas* Strains

**DOI:** 10.1002/mbo3.70316

**Published:** 2026-05-28

**Authors:** Livia Jerjen, Floriane L'Haridon, Laure Weisskopf

**Affiliations:** ^1^ Department of Biology University of Fribourg Fribourg Switzerland; ^2^ Food Research and Innovation Center University of Fribourg Fribourg Switzerland

**Keywords:** biocontrol, *Phytophthora infestans*, potato, *Pseudomonas*, pyoverdine, siderophores

## Abstract

The oomycete *Phytophthora infestans* is a detrimental pathogen currently controlled by massive applications of synthetic pesticides. In view of the pesticides' toxicity, biological control represents an attractive alternative to fight this pathogen. *Pseudomonas* strains are known to produce diverse specialized metabolites conferring protection against several crop diseases. Next to molecules causing direct pathogen inhibition, such as antibiotics and toxins, siderophores are considered important mediators of competitive inhibition of pathogens by *Pseudomonas*. However, whether siderophore production plays any role in the biocontrol of *P. infestans* has not yet been investigated. In this study, we focused on two *Pseudomonas* strains, *Pseudomonas donghuensis* R32 and *Pseudomonas chlororaphis* R47, both producing the siderophore pyoverdine, and previously characterized as inhibitors of *P. infestans*. The aim of this study was to evaluate the role of pyoverdine in the inhibition of *P. infestans* by both strains. For this purpose, we created pyoverdine‐deficient mutants by knocking‐out *pvdE*, the periplasmic ferribactin exporter, in both wild‐types and HCN‐deletion mutants. These mutants were then tested for activity against *P. infestans* in different in vitro assays as well as in leaf discs. Our results indicate that pyoverdine plays no role in the inhibition of *P. infestans* by *P. donghuensis* R32 and only a minor role in *P. chlororaphis* R47, which was observed solely in leaf disc assays. Nevertheless, our findings demonstrate that iron competition plays an important role in the antagonistic effect of *P. donghuensis* R32 on *P. infestans* and suggest the involvement of another siderophore in the competition for iron.

## Graphical abstract

*Pseudomonas* strains R32 and R47 inhibit *Phytophthora infestans*, which causes late blight in potato. To assess the importance of pyoverdine, a high‐affinity siderophore both strains produce, in *P. infestans* inhibition, we produced pyoverdine‐deficient mutants and tested their antagonistic activity at different developmental stages of the pathogen.

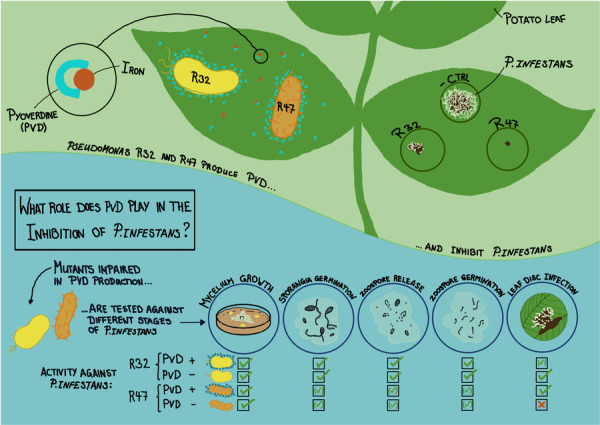

**Abbreviations**
7HT: 7‐hydroxytropolone
ABC: ATP‐binding cassette
BGC: biosynthesis gene cluster
CAS: chrome azurol S
FUR: ferric uptake regulator
HCN: hydrogen cyanide
KO: knock‐out
NRPS: nonribosomal peptide synthetase

## 1 Introduction

Crops are exposed to a variety of pathogens that can cause severe yield losses. In potato in particular, *Phytophthora infestans* stands out as a challenging pathogen worldwide (Kamoun et al. 2015). This oomycete, causing late blight in *Solanaceae* crops, can destroy a potato field in as few as 10–15 days (Rakotonindraina et al. 2012). It is considered the most threatening potato pathogen due to its rapid evolution, high adaptation capacity and fast asexual reproduction cycle involving two types of spores: sporangia that germinate directly under warmer conditions, and motile zoospores that are released from sporangia when temperatures are colder (Fry et al. 2015). Current crop protection practices heavily rely on copper‐based and synthetic pesticides, the latter being not only toxic but also prone to resistance development in such a rapidly evolving pathogen as *P. infestans* (Leesutthiphonchai et al. 2018). In contrast, biocontrol agents, meaning microorganisms used to protect crops, are less likely to trigger resistance development in pathogens due to their multitarget modes of action (Ivanov et al. 2021). In bacteria, these modes of action can encompass induction of plant resistance, or more direct mechanisms like antibiosis and competition for resources and space (Wang and Long 2023). The latter also includes the competition for iron, which, despite its abundance in the environment, is difficult to acquire from soil due to its insolubility in the ferric state (Owen and Ackerley 2011). Microorganisms are able to produce iron‐scavenging molecules, called siderophores, that allow them to take up the insoluble ion from the soil and make them great competitors for iron (Schalk 2008), a feature that has been shown to play an important role in microbial competition (Butaitė et al. 2018) and specifically for biocontrol of plant pathogens (Duijff et al. 1993; Gu et al. 2020; Liu et al. 2021; Ran et al. 2005).

Previous research in our group has focused on two *Pseudomonas* strains showing promise for the biological control of late blight: *Pseudomonas donghuensis* R32 and *Pseudomonas chlororaphis* R47. Both strains showed strong inhibitory activity on different developmental stages of *P. infestans* (mycelium, sporangia, and zoospores) (Anand et al. 2020, 2023; Hunziker et al. 2015). A comparative genomics approach identified putative biocontrol traits and mechanisms in these two strains (De Vrieze et al. 2018, 2020; Hunziker et al. 2015), among which are the production of phenazines by *P. chlororaphis* R47, and the production of hydrogen cyanide (HCN) and pyoverdine by both strains. The role of HCN in both strains' anti‐*Phytophthora* activity has been investigated previously (Anand et al. 2020), and was shown to affect mycelial growth but not spore germination, nor disease progression in leaf discs. In contrast, the importance of pyoverdine for the anti‐*Phytophthora* activity has not been evaluated yet, although it was reported previously to be relevant for biocontrol (Duijff et al. 1993; Gu et al. 2020; Liu et al. 2021; Sass et al. 2018; Trapet et al. 2016).

Pyoverdine was first described as a siderophore in 1978 (Meyer and Hornsperger 1978). Since then, many studies revealed its importance in iron scavenging and as a virulence factor in different biological contexts (Chatterjee et al. 2017; Díaz‐Pérez et al. 2023; Liu et al. 2021; Matilla et al. 2007; Sass et al. 2018; Taguchi et al. 2010). The biosynthesis of pyoverdines is known to vary across *Pseudomonas* species (Lamont and Martin 2003) and strains and is reflected by a large variety of different structures (Visca et al. 2007). Typically, pyoverdines consist of a dihydroxyquinoline fluorophore, a variable acyl side chain attached to its 3‐amino group and a strain‐specific peptide backbone which is bound to the C_1_‐carboxyl group of the fluorophore (Ringel and Brüser 2018). Biosynthesis starts in the cytoplasm with the assembly of ferribactin, pyoverdine's precursor, by mostly 3–4 nonribosomal peptide synthetases (NRPSs) and an array of auxiliary enzymes (Ringel and Brüser 2018; Schalk and Guillon 2013; Visca et al. 2007). The exact number and identity of NRPS can vary depending on the strain, but PvdL stands out as the only conserved one across all species (Ravel and Cornelis 2003) and as the starting point of the synthesis (Manko et al. 2024). Ferribactin is then transported into the periplasm by PvdE, an ATP‐binding cassette transporter sitting on the inner membrane (Yeterian et al. 2010). There, the precursor undergoes a diacylation by PvdQ (Wurst et al. 2014), and finally, the characteristic fluorophore is formed. This nonmatured pyoverdine undergoes modifications until it is finally exported by a tripartite transporter, PvdRT‐OpmQ, where it can act as an iron scavenger. Ferripyoverdine is again imported into the periplasm by the outer membrane receptor FpvA, which is specific to the strain‐specific structure of pyoverdine. It is then detached from the iron ion and is exported again outside the cell (Ghssein and Ezzeddine 2022; Ringel and Brüser 2018).

The synthesis of pyoverdine is mainly regulated by iron availability through the cytoplasmic Fur regulator. Under low iron conditions, the anti‐sigma factor FpvR, and the sigma factors FpvI and PvdS are no longer repressed by Fur. While FpvR is inhibiting the two sigma factors, which themselves activate pyoverdine synthesis and uptake genes, it autoproteolyses upon encounter with iron‐bound FpvA (Edgar et al. 2014). This mechanism ensures positive feedback under iron starvation but also allows shutdown when there is enough iron. It is also known that depending on the strain, pyoverdine synthesis can be regulated by the global regulator GacS/GacA (Ferreiro and Gallegos 2021), varying nutrient availabilities, such as sulfur or phosphor (Delic‐Attree et al. 1997), or other virulence factors, in a complex network of regulation (Ringel and Brüser 2018).

Although siderophores in general, and pyoverdines in particular, have been shown to play a role in the biological control of plant diseases (Liu et al. 2021; Ran et al. 2005), the extent of pyoverdine's contribution to the anti‐*Phytophthora* properties of *Pseudomonas* strains remains unknown. To fill this gap in knowledge, we created pyoverdine mutants in the two *Pseudomonas* biocontrol strains: *P. donghuensis* R32 and *P. chlororaphis* R47. In addition to single knock‐outs (KOs), we also performed double KOs of both HCN and pyoverdine to avoid a putative impact of pyoverdine being masked by the effect of HCN on *P. infestans*. We selected *pvdE*, which encodes the periplasmic ferribactin importer, as a target for mutagenesis, as such a procedure was reported to abolish pyoverdine production in *Pseudomonas aeruginosa* (McMorran et al. 1996; Yeterian et al. 2010). We then characterized the ability of these mutants to inhibit different developmental stages of *P. infestans* in in vitro experiments, as well as their impact on disease progression in leaf discs to quantify the contribution of pyoverdine to the inhibitory effect of both *Pseudomonas* strains on *P. infestans*.

## 2 Material and Methods

### 2.1 Strains and Culture Conditions

*P. donghuensis* R32 and *P. chlororaphis* R47 were originally isolated from the rhizosphere of potato plants (Hunziker et al. 2015). The two wild‐types and their respective mutants were routinely cultured on LB‐Agar plates with 10 µM rifampicin at 28°C. LB agar plates were prepared with 12.5 g·L^−1^ LB Broth Miller (Roth), 10 g·L^−1^ LB Broth Lennox (Roth), and 15 g·L^−1^ of Agar‐Agar‐Kobe I (Roth), which were dissolved in distilled water and autoclaved. Liquid cultures were incubated overnight at 180 rpm at 28°C.

King's B (KB) medium was prepared by mixing 20 g·L^−1^ proteose peptone #3 (Gibco), 1.5 g·L^−1^ K_2_HPO_4_ (Roth), 1.5 g·L^−1^ MgSO_4_·7H_2_O (Roth), 10 mL·L^−1^ glycerol (Reactolab), and 15 g·L^−1^ Agar‐Agar‐Kobe I (Roth) for solid medium, dissolving them in distilled water and autoclaving. *P. infestans* strains Rec01, 44 and 208m2 (green fluorescent protein [GFP]‐tagged strain; Si‐Ammour et al. 2003) were routinely grown at 18°C on V8‐Agar plates. This medium was prepared by diluting V8 100% hot spicy vegetable juice at 100 mL·L^−1^ in distilled water and adding 1 g·L^−1^ of CaCO_3_ (Roth) and 15 g·L^−1^ of agar. Liquid V8 medium was filtered in some experiments with 0.22 µm filters (Millex) after autoclaving to get rid of debris. After a maximum of seven passages on plates, *P. infestans* was passaged on potato tubers to maintain its virulence.

### 2.2 Generation of KO Mutants in *P. donghuensis* R32 and *P. chlororaphis* R47

We followed the same protocol as described in a previous study (Anand et al. 2020) to generate *pvdE* KO mutants in both R32 and R47 wild‐type and ∆*hcn* strains. Please refer to Table S1 for the list of plasmids and strains, and Table S2 for the primers used. The *pvdE* gene was truncated by amplifying two fragments on each gene border and ligating them together. Since for *P. donghuensis* R32, the attempts to amplify the gene fragments for the first cloning step were unsuccessful, the truncated sequence was produced synthetically by Eurofins (Eurofins Scientific 2023). The truncated *pvdE* gene was cloned into the pEMG plasmid, which was electroporated into the respective strains' genomes by homologous recombination. Transformants were selected by kanamycin resistance, and the presence of the truncated gene was confirmed by PCR. In a second step, we got rid of the plasmid backbone by electroporating the transformants with PSW‐2, a plasmid containing the gene coding for the Sce‐I restriction enzyme, which after cutting should leave either the wild‐type (reversed wild‐type), or the truncated *pvdE* gene in the genome. Mutants which regained sensitivity to kanamycin were selected, and the correct genotype was verified by PCR. In a later step, we confirmed the phenotype (loss of pyoverdine) by fluorescence measurements.

### 2.3 Growth Curves and Pyoverdine Measurements

Overnight bacterial cultures in LB were centrifuged at 5000 rpm, the supernatant removed, and the pellet resuspended in 0.9% NaCl (Roth). After a second centrifugation step, the washing supernatant was removed and resuspended in the same solution. OD_600_ was adjusted to 1, and in a 96‐well plate, we mixed 5 µL of the bacteria (or 0.9% NaCl for the negative control) with 195 µL of either liquid LB, KB, or filtered V8. For experiments with iron addition or chelation, the mixes were supplemented with FeCl_3_·6H_2_O (Merck) in different concentrations (27, 12, or 1 mg·L^−1^) or 1 mM ethylenediaminetetraacetic acid (EDTA) (Roth), respectively. We performed triplicates for each sample. The plate was incubated for 48 h in a Cytation 5 cell imaging reader (Agilent) with continuous shaking at 28°C. Every hour, absorbance at 600 nm and pyoverdine fluorescence (excitation at 405 nm and emission at 460 nm) were measured. For incubations at 21°C, we did so in a shaking incubator and measured only specific time points in the Cytation 5, with the same parameters as above.

To compare pyoverdine production of the different genotypes at specific time points, as Gaussian distribution and heteroscedasticity were given, statistical analysis was performed using a one‐way analysis of variance (ANOVA), followed by Tukey's test, where *p < 0.05, **p < 0.01, and ***p < 0.001. For the assay with FeCl_3_ supplementation, the assumptions of normal distribution and heteroscedasticity were not met, so we performed a mixed‐effect model analysis with Geisser–Greenhouse correction, followed by Tukey's test.

### 2.4 Siderophore Detection With Chrome Azurol S (CAS)

CAS media were prepared by mixing 10 mL of iron solution (270 mg·L^−1^ FeCl_3_·6H_2_O [Merck] and 1% HCl 1 M [Fisher Chemicals] in distilled water) with 50 mL of solution 1 (1.21 g·L^−1^ CAS [Kodak] in distilled water). A 40 mL of solution 2 (1.8225 g·L^−1^ hexadecyltrimethylammonium bromide [HDTMA, Sigma] dissolved in distilled water) was added to the first mix, and the final CAS solution was autoclaved. KB‐Agar was prepared as described above, and before autoclaving, 33.6 g·L^−1^ of PIPES PUFFERAN (Roth) was added, the pH was adjusted to 6.8 with NaOH, and then 15 g·L^−1^ of agar was added. KB medium was autoclaved, then mixed with the CAS solution in a ratio of 9:1. Overnight bacterial cultures in LB were centrifuged at 5000 rpm, the supernatant removed, and the pellet resuspended in 0.9% NaCl (Roth). After a second centrifugation step, the washing supernatant was removed, and the pellet was resuspended in the same solution. OD_600_ was adjusted to 1, and 10 µL of the bacterial suspension was pipetted onto CAS‐KB plates before incubating for 48 h at 28°C.

### 2.5 HCN Detection

In all, 50 g·L^−1^ of copper(II)‐ethyl acetoacetate (Aldrich) and 50 g·L^−1^ 4,4‐methylenbis(*N*,*N*‐dimethylaniline) (Sigma‐Aldrich) were dissolved in chloroform (Fisher Chemicals). This solution was then pipetted onto pieces of filter paper (Whatman) that had been cut beforehand. The paper was dried overnight under a fume hood and then stored in a glass container wrapped in aluminum foil at 4°C. Split Petri dishes (Sarstedt) were filled on one side with LB‐Agar and left empty on the other side. Bacteria (10 µL) at OD_600_ = 1 were pipetted onto the LB‐Agar and a detection paper placed into the empty dish side. Plates were sealed with parafilm and incubated at 30°C. Pictures were taken at 24 and 48 h after incubation.

### 2.6 In Vitro Dual Assays

Experiments were performed on plates filled with V8‐Agar, which in some experiments was supplemented with FeCl_3_ (27, 12, or 1 mg·L^−1^). Overnight cultures of bacteria in LB were rinsed in 0.9% NaCl (Roth) as described above and adjusted to OD_600_ = 1 in the same solution. Three drops of 10 µL of bacterial suspension (or 0.9% NaCl for the negative control) were carefully applied symmetrically onto a V8‐Agar plate near the border. A plug of *P. infestans* Rec01 culture was then placed in the middle of the plate. Sealed plates were incubated at 21°C for 12–14 days. Four replicate plates per bacterial strain and six replicate plates for the control (*P. infestans* growing alone) were used per experiment. For the analysis, the area of growth of the pathogen's mycelium and the area of the bacterial colony were measured with ImageJ (Schneider et al. 2012). The percentage of growth inhibition caused by the bacteria was calculated with the following formula: Inhibition % = 100 ∗ *Ai*/(*AC* − *Ab*), with inhibition area *Ai* = *AC* − *Ab* − *At*, where *AC* = pathogen area in control plates, *Ab* = bacterial area, and *At* = pathogen area. Note that the inhibition area was calculated considering that the bacterial area is not available anymore for the pathogen because of space competition (*AC* − *Ab*). Gaussian distribution and heteroscedasticity were not given, so we performed a mixed‐effect model analysis with Geisser–Greenhouse correction, followed by Tukey's test, where *p < 0.05, **p < 0.01, and ***p < 0.001.

### 2.7 Spore Assays

We performed zoospore release, zoospore germination and sporangia germination assays, and for all three types, an overnight liquid culture of bacteria in LB was rinsed in 0.9% NaCl (Roth) as described above and adjusted to OD_600_ = 1 in filtered V8. Bacterial suspension (50 µL) was transferred into flat‐bottom 96‐well plates (Costar). The procedure for each assay differed from here, as detailed below.

For the zoospore germination assay, 50 µL of 0.9% NaCl was added to the bacterial suspension. Then, 10 mL ice‐cold, sterile water was poured onto 2–3‐week‐old *P. infestans* cultures on V8‐agar plates, which were placed at 4°C in the dark for 2 h. After this cold shock, the plates were placed at 18°C for 30 min. Zoospores were then collected by aspirating the water from the mycelium surface with a pipette, and their concentration was adapted to 3 × 10^4^ zoospores·mL^−1^. Zoospore solution (40 µL) was added to the wells. The sealed plates were then incubated in the dark at 18°C for 3–4 h.

For the zoospore release assay, sporangia were collected by adding 2–3 mL of sterile water to a 2–3‐week‐old *P. infestans* plate and scratching off the mycelium from the agar with a glass slide. The mycelium was washed through a filter with sterile water into a 15 mL Falcon tube (Cellstar), so that sporangia were separated from the mycelium. The sporangia solution was then adapted to 3 × 10^4^ sporangia·mL^−1^, and 40 µL was added into wells. Ice‐cold sterile water (50 µL) was added into the wells, and the plate was placed at 4°C in the dark for 2 h, then for 30 min at 18°C, still in the dark.

For the sporangia germination assays, 50 µL of 0.9% NaCl was added to the wells. Sporangia were collected as described above, and 40 µL of the final solution of 3 × 10^4^ sporangia·mL^−1^ was added before incubating in humid, dark conditions for 24 h.

After the respective incubation times, pictures were taken with the Cytation 5 (Agilent), and then percentages of germination for sporangia and zoospores were calculated by counting germinated versus nongerminated spores (total spores per replicate ca. 200 spores), and zoospore release percentage was calculated by counting full versus empty sporangia, counting the same number of spores as above per picture. All experiments were performed in technical triplicate. For statistical analysis, we pooled together results from four independent experiments. Since we compared percentages of different categories and could not assume normal distribution and heteroscedasticity, we used a Kruskal–Wallis test followed by Dunn's multiple comparisons test performed either on GraphPad Prism or R Studio.

For sporangia germination, we measured the length of germ tubes additionally with ImageJ in two experiments with three technical replicates each and performed an ANOVA with Tukey's post‐hoc test for statistical analysis. This was done under the assumption of a Gaussian distribution and heteroscedasticity.

### 2.8 Leaf Disc Assays

Potato plants from the Bintje cultivar were cultivated in a greenhouse for 6–7 weeks. Leaf discs were cut and placed abaxial side up onto a 0.8% water agar plate (five discs per plate) the day before inoculation and infection. We analyzed four plates per bacterial treatment and negative control (20 leaf discs). One leaf disc per plant was used as a noninfected control. Bacteria were cultured overnight in LB, washed in 0.9% NaCl and resuspended at OD_600_ = 2 in the same solution. Zoospores were harvested from 2‐week‐old *P. infestans* cultures as described above. The zoospore solution was adjusted to 6–8 × 10^4^ spores·mL^−1^. The bacterial suspension and zoospore solution were mixed in a 1:1 ratio, and 10 µL of this mixture was applied onto each leaf disc. Plates were then placed into a transparent, closed box with high humidity at 21°C with a light/dark cycle for a maximum of 6 days. Pictures were taken at 4 and 5 dpi. Lesion development was scored at 4 dpi from 1 (no lesion) to 5 (full necrosis), and mycelium development was scored at 5 dpi from 1 (no mycelium) to 5 (fully covered by mycelium). Since we compared different categories and thus could not assume normal distribution and heteroscedasticity, we used a Kruskal–Wallis test followed by Dunn's multiple comparisons test for both scores separately.

### 2.9 Survival on Leaf Discs

Leaf discs were prepared as described above. Bacteria were cultured overnight in LB, washed in 0.9% NaCl and resuspended at OD_600_ = 1 in the same solution (the final concentrations used in infected leaf discs also). The bacteria (10 µL) were pipetted onto the center of the leaf disc and incubated during 7 days under the same conditions as described above. Per treatment, three leaf discs were collected and placed in an Eppendorf tube with 1 mL of sterile, distilled water. Each sample was vortexed for 10 s, followed by a 3‐min sonication. This was repeated a second time and followed by a final 10‐s vortexing step. Then, a dilution series up to 10^−6^ was performed, and a 10‐µL drop of each dilution was pipetted onto an LB plate supplemented with rifampicin (Roth). The plates were tilted carefully to let the drops run down the plate, and then incubated overnight at 28°C. Thereafter, colony‐forming units were counted.

## 3 Results

### 3.1 Deletion of *pvdE* Leads to Decreased Secretion of Pyoverdine in Both *P. donghuensis* R32 and *P. chlororaphis* R47

To evaluate the impact of siderophore production on the anti‐*Phytophthora* activity of *P. donghuensis* R32 and *P. chlororaphis* R47, pyoverdine mutants were generated by deleting the *pvdE* gene in both wild‐type and HCN‐deletion mutant backgrounds (Anand et al. 2020). This mutation did not impair the strains' growth in the rich medium LB, and only slightly in iron‐poor KB, while only the double mutant of R32 showed a reduced growth in V8 (Figure S1). When supplementing these media with FeCl_3_, it equalized growth conditions between the mutant and wild‐type genotypes of both strains for the media with lower availability of iron, KB and V8. For the already iron‐rich medium LB, adding external iron even led to a growth advantage for both *pvdE* single and double mutants of *P. donghuensis* R32. On the other hand, when supplementing these media with the iron chelator EDTA, we observed a strongly reduced growth of all pyoverdine mutant genotypes in LB, and a reduced growth of the same in KB and V8, especially for the double mutant (Figure S1). We suppose that the iron in the different media is not reacting in the same way to the addition of EDTA, because it might already be complexed in V8 and KB, but not in LB, and thus in the latter it might be completely captured by the added EDTA, and therefore less accessible for the pyoverdine mutants.

**Figure 1:**
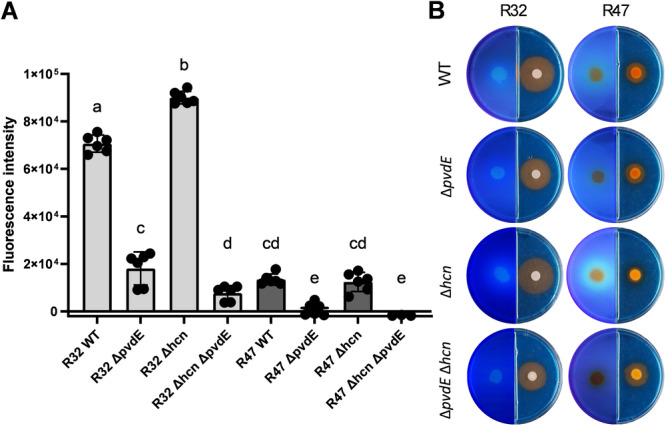
Siderophore detection in *Pseudomonas donghuensis* R32 and *Pseudomonas chlororaphis* R47. (A) Pyoverdine levels were measured by fluorescence emission in 48 h old bacterial cultures grown in KB. Negative control values were subtracted from sample values first, after which sample values were normalized to OD_600_ = 1. Bars represent the mean of two biological replicates with three technical replicates each. Statistical analysis was performed using a one‐way ANOVA, followed by Tukey's test. (B) Pictures of the siderophore detection assays on plates. On the left side of each plate, bacteria were grown on KB medium for 48 h and then pictures were taken under ultraviolet light to visualize fluorescent molecules (mainly fluorescent siderophores). On the right side of the plates, bacteria were grown on KB‐CAS medium for 48 h. Siderophore diffusion is visualized by an orange halo. The pictures shown are representative examples of two technical replicates. ANOVA, analysis of variance; CAS, Chrome Azurol S; KB, King's B; WT, wild type.

As expected, pyoverdine levels as revealed by fluorescence emission (Kang and Kirienko 2017) were strongly reduced in single and double mutants of both strains in KB (Figure 1A) and filtered V8 (Figure S2), although production was not completely abolished in *P. donghuensis* R32. Higher pyoverdine emission was observed in R32 than in R47, and in single ∆*hcn* mutants than in the respective wild‐type strains, as previously reported (Anand et al. 2023). This was visible in the pyoverdine measurement in liquid cultures of *P. donghuensis* R32 (Figure 1A) and in the ultraviolet (UV) fluorescence emitted on solid culture plates for *P. chlororaphis* R47 (Figure 1B). To gain a broader view of siderophore production beyond the measurement of pyoverdine and visualization of fluorescent molecules, we grew the eight genotypes on CAS plates, on which siderophore‐mediated iron depletion can be visualized by an orange halo. In contrast to our expectations, we observed no clear correlation between pyoverdine fluorescence measurements and the size of the orange halo, which suggests that this halo is mediated by the secretion of one or more other siderophore(s).

**Figure 2:**
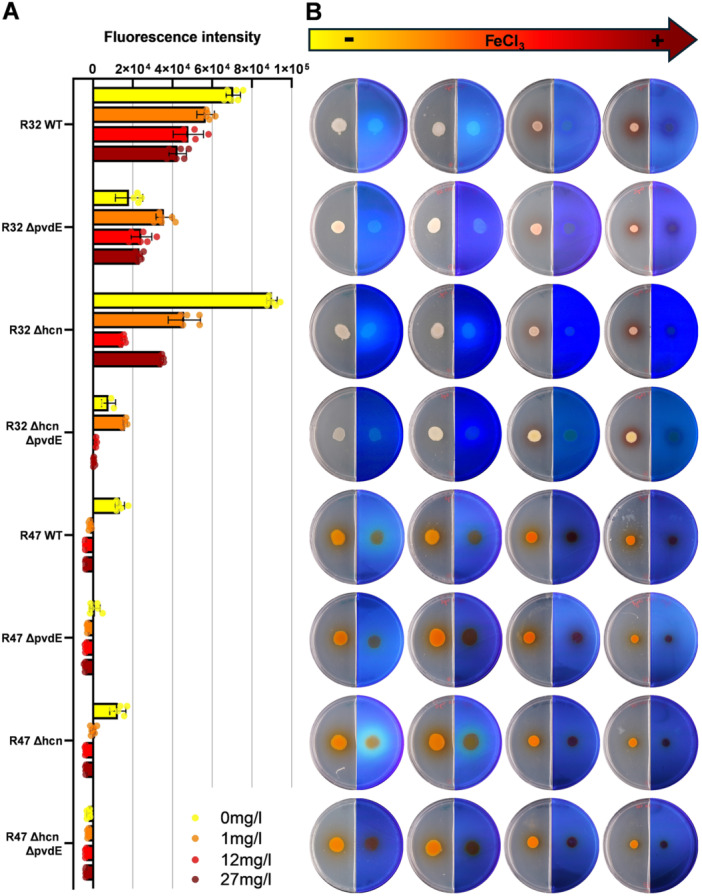
Pyoverdine detection in *Pseudomonas donghuensis* R32 and *Pseudomonas chlororaphis* R47. (A) Pyoverdine levels were measured by fluorescence emission in 48 h old bacterial cultures grown in KB medium supplemented with different FeCl_3_ concentrations (0, 1, 12, and 27 mg·L^−1^). Negative control values were subtracted from sample values first and then normalized to OD_600_ = 1. Bars represent the mean of two biological replicates with three technical replicates each. See Table S3 for statistical analysis. (B) Pictures of pyoverdine fluorescence on plates (0 mg·L^−1^ of iron on the left, increasing iron concentrations to the right). Bacteria were grown on KB medium for 48 h. On the left side, pictures were taken under normal light. On the right side, we can see the exact same cultures, but pictures were taken under ultraviolet light to visualize fluorescent molecules, mainly fluorescent siderophores. WT, wild type.

As expected, pyoverdine production correlated with iron deficiency, although differences were observed between the two strains: *P. donghuensis* R32 gradually decreased its pyoverdine production with increasing iron concentrations but still produced detectable amounts at the highest iron supply tested (27 mg·L^−1^), while *P. chlororaphis* R47 stopped producing pyoverdine already when supplied with 1 mg·L^−1^ iron in its growth medium (Figure 2A and Table S3).

### 3.2 Iron Supply Decreased the Ability to Restrict *P. infestans* Mycelial Growth in *P. donghuensis* R32, but Not in *P. chlororaphis* R47

**Figure 3:**
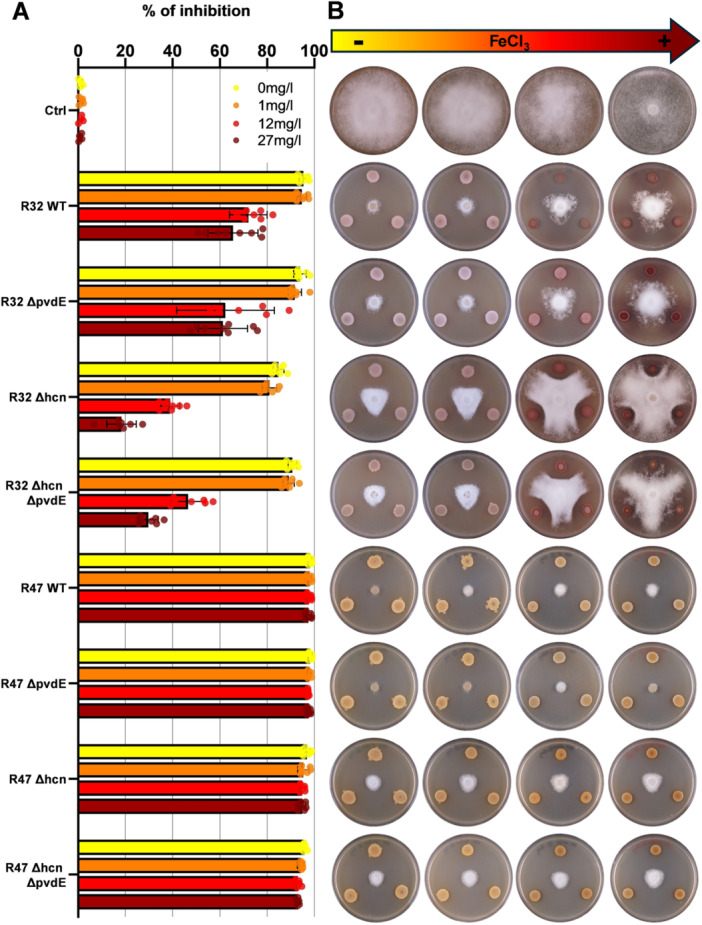
Mycelial inhibition assay on media with different iron concentrations. *Phytophthora infestans* and the different genotypes of *Pseudomonas donghuensis* R32 and *Pseudomonas chlororaphis* R47 were grown for 14 days on V8 medium supplemented with different FeCl_3_ concentrations (0, 1, 12, and 27 mg·L^−1^). (A) The mean inhibition percentage was calculated relative to the negative control, which represents 0% of inhibition. Averages of two biological replicates with four technical replicates each are shown. See Table S4 for statistical analysis. (B) Representative pictures of the inhibition assays that were used to generate the data in (A) (0 mg·L^−1^ of iron on the left, increasing iron concentrations to the right). WT, wild type.

In V8 medium without iron supplementation, we observed no significant loss of activity in single ∆*pvdE* mutants compared with the wild‐type strains (Figure 3A,B and Table S4). While all *P. chlororaphis* R47 mutants kept the full inhibition potential of the wild‐type, slight modulations were observed in *P. donghuensis* R32, with a small but significant decrease of activity in the single ∆*hcn* mutant and a partial rescue of the wild‐type phenotype in the double mutant (Figure 3A and Table S4). Adding increasing concentrations of iron did not impair *P. chlororaphis* R47's ability to inhibit mycelial growth, suggesting that competition for iron is not involved in this process. In contrast, both wild‐type and mutant strains of *P. donghuensis* R32 lost part of their activity with increasing iron supply, suggesting that part of the observed inhibition potential was due to iron deprivation of *P. infestans*. This was most visible in the absence of HCN. However, ∆*pvdE* mutants did not show a more severe activity loss than their corresponding controls (wild‐type vs. single ∆*hcn* mutant) as could have been expected if pyoverdine had played a role in iron acquisition. On the contrary, the double KO seemed to regain part of the wild‐type's inhibitory potential independently of iron supplementation, suggesting the putative upregulation of a yet unknown factor leading to mycelial restriction when both HCN and pyoverdine are no longer produced.

It was striking to observe that *P. donghuensis* R32 and its respective mutants were accumulating and secreting a reddish molecule when growing on plates supplemented with high iron concentrations (Figure 3B), a phenomenon that was the most intense in the single HCN mutant in *P. donghuensis* R32. In *P. chlororaphis* R47, no secretion of this molecule could be observed, only a slightly darker coloration in the HCN mutant and double mutant at the highest iron concentrations (Figure 3B). A phenotype visible in both strains was the decreasing colony diameter with increasing iron concentrations, which could be due to a higher agar density mediated by iron addition, and was not linked to toxicity, as cell numbers revealed by CFU counting were not reduced even at the highest iron dose (Figure S3).

### 3.3 Mutants Deprived of Both HCN and Pyoverdine Lost Their Spore Inhibition Potential in *P. donghuensis* R32, but Not in *P. chlororaphis* R47

**Figure 4:**
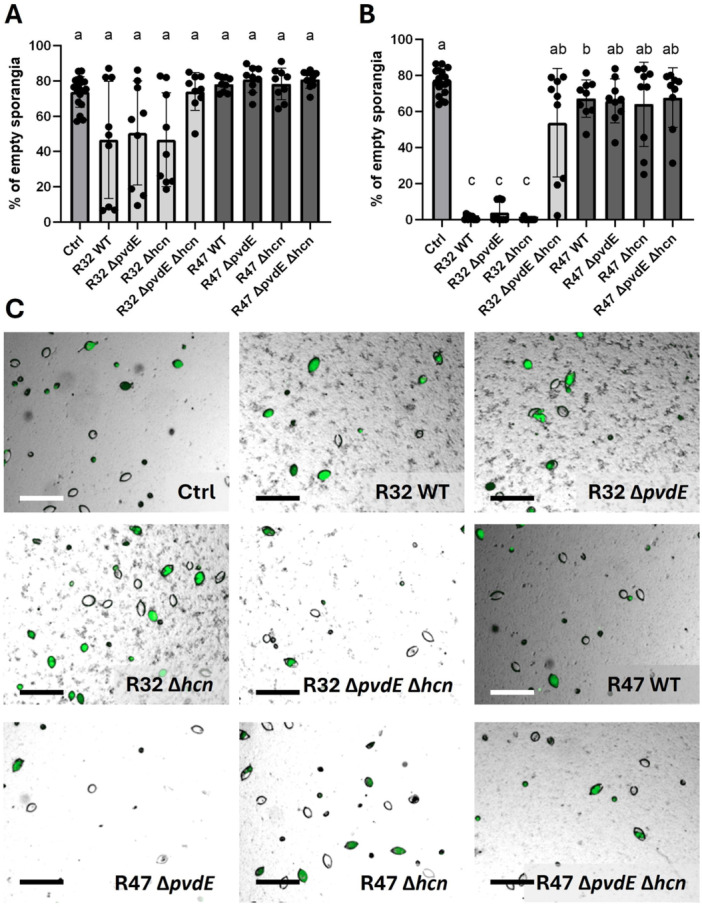
*Phytophthora infestans* zoospore release in the presence of *Pseudomonas donghuensis* R32 and *Pseudomonas chlororaphis* R47. (A, B) After being exposed to cultures of the different strains at OD_600_ = 0.25 (A) or OD_600_ = 0.5 (B) during 2.5 h, the numbers of empty sporangia (successful release) and full sporangia (inhibited release) were counted, and the number of empty sporangia was expressed as a percentage of total sporangia. Bars represent the average of three biological replicates with three technical replicates each. Statistical analysis was performed using a Kruskal–Wallis multiple comparisons test, followed by Dunn's test. (C) Representative pictures of the assay at OD_600_ = 0.25. Zoomed cut‐outs are shown, and size bars correspond to 100 µM. WT, wild type.

**Figure 5:**
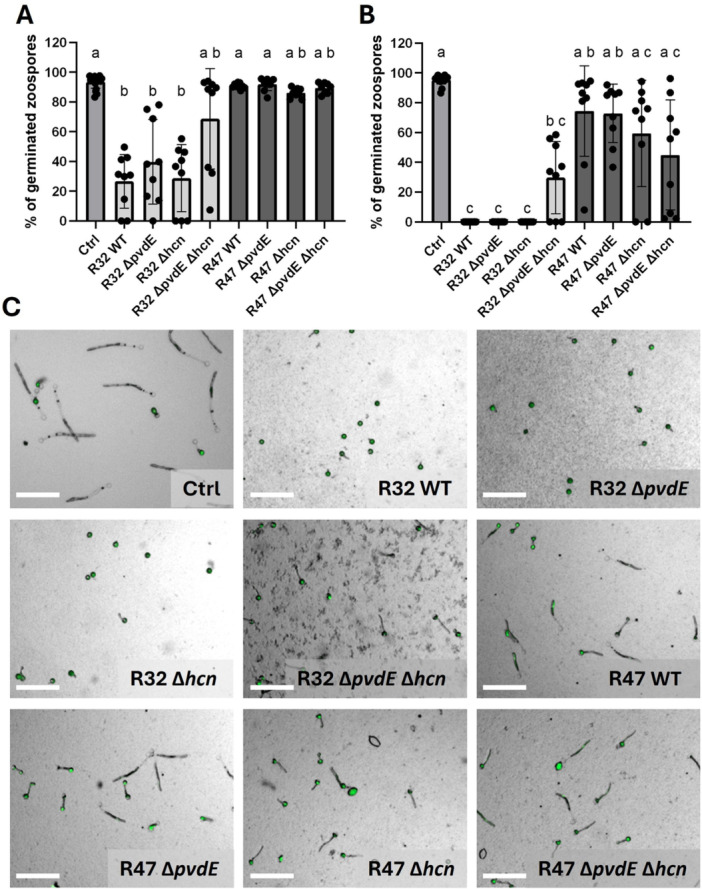
*Phytophthora infestans* zoospore germination in the presence of *Pseudomonas donghuensis* R32 and *Pseudomonas chlororaphis* R47. (A, B) After being exposed to cultures of the different strains at OD_600_ = 0.25 (A) or OD_600_ = 0.5 (B) during 4 h, the numbers of germinated and nongerminated zoospores (inhibition) were counted, and the number of germinated zoospores was expressed as a percentage of total zoospores. Bars represent the mean of three biological replicates, with three technical replicates each. Statistical analysis was performed using a Kruskal–Wallis multiple comparisons test, followed by Dunn's test. (C) Representative pictures of the assay at OD_600_ = 0.25. Zoomed cut‐outs are shown, and size bars correspond to 100 µM. WT, wild type.

**Figure 6:**
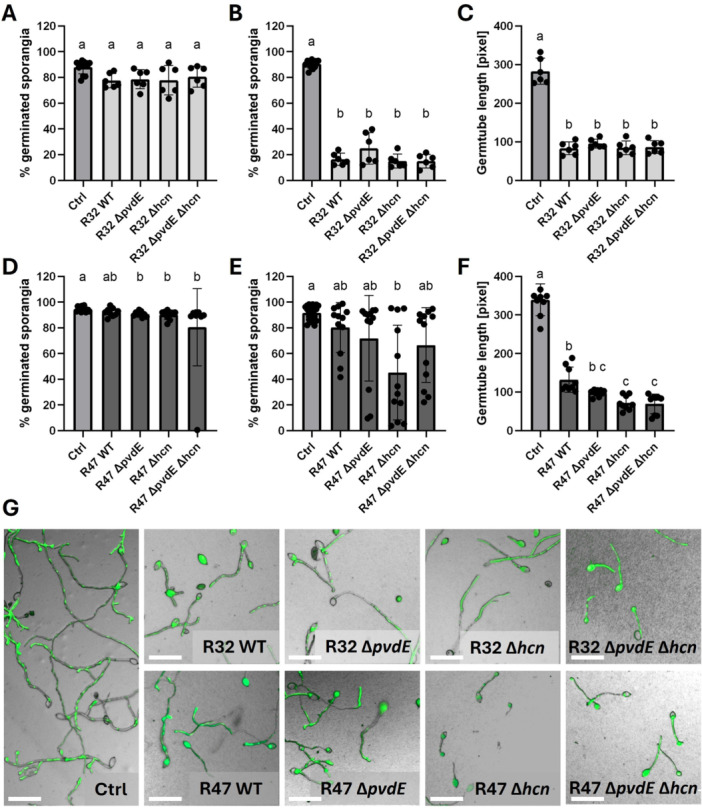
*Phytophthora infestans* GFP sporangia germination in the presence of *Pseudomonas donghuensis* R32 and *Pseudomonas chlororaphis* R47. After being exposed to the different strains for 24 h, we counted germinated and nongerminated sporangia, and expressed the number of germinated sporangia as a percentage of total sporangia. Bars represent the mean of two biological replicates with three technical replicates each. Statistical analysis for germ tube length was performed with a one‐way ANOVA followed by Tukey's test. For the percentage of germinated sporangia, we used a Kruskal–Wallis multiple comparisons test, followed by Dunn's test. (A) Percentages of germinated sporangia exposed to R32 at OD_600_ = 0.01. (B) Percentages of germinated sporangia exposed to R32 at OD_600_ = 0.05. (C) The length of the germ tubes exposed to R32 at OD_600_ = 0.01 was measured in pixels. (D) Percentages of germinated sporangia exposed to R47 at OD_600_ = 0.25. (E) Percentages of germinated sporangia exposed to R47 at OD_600_ = 0.5. (F) The length of the germ tubes exposed to R47 at OD_600_ = 0.25 was measured in pixels. (G) Representative pictures of the assays at OD_600_ = 0.01 for R32 and OD_600_ = 0.25 for R47 genotypes. Zoomed cut‐outs are shown, and size bars correspond to 100 µM. GFP, green fluorescent protein; WT, wild type.

In addition to mycelial growth inhibition, we analyzed whether pyoverdine would play a role in the inhibition of zoospore release (Figure 4), zoospore germination (Figure 5), and sporangia germination (Figure 6). As spore physiology shows higher variability between pathogen strains than mycelial growth, we analyzed three different *P. infestans* genotypes. Similar results were obtained for the three strains (see Figures S4–S6), hence we only present the results obtained with the GFP‐labeled strain in Figures 4, 5, 6.

In general, *P. chlororaphis* R47 showed very little spore inhibition potential compared with *P. donghuensis* R32, which led to strong inhibition even when applied at low cell densities, whereby the results were quite variable at these low densities. In both zoospore release and germination, no significant change of activity was observed in the mutant genotypes of *P. chlororaphis* R47 compared with the wild‐type at both tested concentrations (Figures 4, 5 and 5A,B). Although not significant, we could still observe a tendency towards increased antagonistic activity for the *P. chlororaphis* R47 HCN single mutant and the double mutant in the zoospore germination (Figure 5A,B).

In *P. donghuensis* R32, both single mutants kept their full inhibition potential, and a loss of inhibition was observed only for the double mutant. For zoospore release, the effect that was only a tendency at lower bacterial concentration (Figure 4A) became a significant loss of inhibition at the higher concentration (Figure 4B), where sporangia treated with the double KO almost reached the same level of zoospore release as those of the water control. Regarding zoospore germination, at both tested concentrations, the difference between the double mutant and all other *P. donghuensis* R32 genotypes was not statistically significant due to high variability between replicates, but the same tendency towards reduced activity was visible (Figure 5A,B). Since no loss of inhibition was detected for either of the single mutants, this result suggests an interplay or a redundant effect of both biocontrol traits.

Sporangia germination yielded a contrasting picture, in which no difference between wild‐type and mutants was observed, neither in R32 nor in R47, for germination percentages (Figure 6A,B,D,E, respectively). When looking at germ tube length, neither loss of pyoverdine nor loss of HCN affected the inhibition ability of *P. donghuensis* R32 (Figure 6C), while for *P. chlororaphis* R47, both single and double ∆*hcn* mutants showed higher activity than the wild‐type, suggesting the upregulation of other active compounds in the absence of HCN (Figure 6F).

### 3.4 Dual Loss of HCN and Pyoverdine Decreased Activity on Leaf Discs in *P. chlororaphis* R47 but Increased Activity in *P. donghuensis* R32

**Figure 7:**
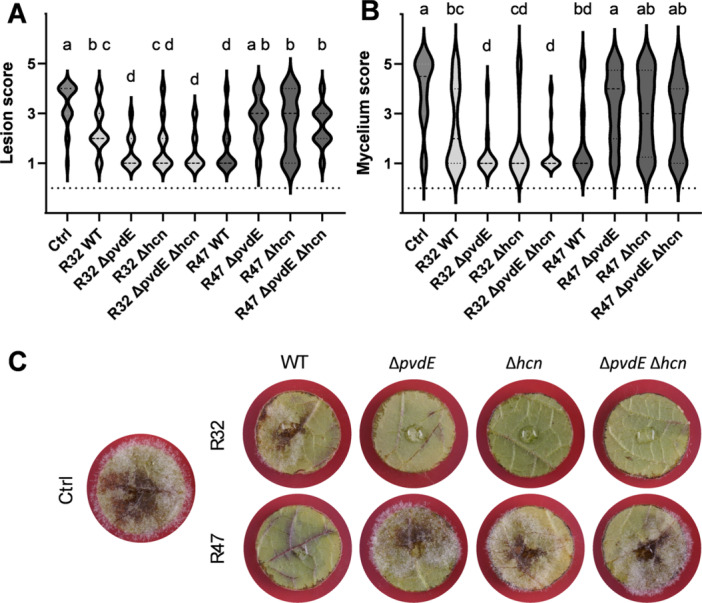
Leaf disc infection assay in the presence of *Pseudomonas donghuensis* R32 and *Pseudomonas chlororaphis* R47. Leaf discs from the Bintje cultivar were infected with *Phytophthora infestans* GFP zoospores and, at the same time, inoculated with different genotypes of R32 and R47. Each treatment was tested on 20 individual leaf discs. (A) Lesion formation was scored from 1 (no lesion) to 5 (full necrosis) at 4 dpi. Statistical analysis was performed with a Kruskal–Wallis test followed by Dunn's test. (B) Mycelium formation was scored from 1 (no mycelium) to 5 (extensive mycelium) at 5 dpi. The same statistical analysis as for the lesion score was performed. (C) Pictures from one representative leaf disc per treatment are shown. GFP, green fluorescent protein; WT, wild type.

Since infection of the leaf discs was performed with zoospores in direct contact with the bacteria, we expected to have similar results as in the zoospore germination assays. Surprisingly, the results showed opposite trends compared with the in vitro experiment, underlining the differing conditions of in vitro and in planta assays (Figure 7). In *P. donghuensis* R32, the *pvdE* single mutant displayed a significant increase of protection against *P. infestans* compared with the wild‐type, both when looking at lesion size (Figure 7A) or mycelium development (Figure 7B). The double mutant was also significantly more protective compared with the wild‐type, but not when compared with ∆*hcn*, since this mutant already showed a trend towards higher protection. These findings suggest an upregulation of one or several other biocontrol traits in the absence of HCN and pyoverdine. In the case of *P. chlororaphis* R47, we observed a complete loss of protective activity in all mutant strains compared with the wild‐type. In this case, HCN and pyoverdine appeared to be involved in the protection of leaf discs against *P. infestans*.

To rule out that different protective activities might be due to differential survival on the leaf discs over the course of the infection experiment, we quantified the survival of the different strains after one week on the leaf discs. All genotypes not only survived for seven days, but also were even able to grow (Figure S7). There was no significant difference in survival between the genotypes, although there was a slight tendency for pyoverdine single and double mutants in R32 to multiply the least.

## 4 Discussion

To assess the importance of pyoverdine in the biocontrol activity of two *Pseudomonas* strains against *P. infestans*, we knocked out the *pvdE* gene in both wild‐type and HCN‐deletion mutants. Although Yeterian et al. (2010) reported to prevent pyoverdine production and accumulation in *P. aeruginosa* by knocking out this gene, we could only achieve the same in one of our two strains. In *P. donghuensis* R32, both the single *pvdE* mutant and the double mutant still displayed decreased but measurable pyoverdine levels, corresponding approximately to the wild‐type levels in *P. chlororaphis* R47 (Figure 1A). While it was still significantly reduced (ca. sevenfold in KB) compared with the wild‐type levels in R32, the question arises as to why we could not achieve a complete depletion of pyoverdine in this strain. While another tripartite pyoverdine secretion pump was found in *Pseudomonas putida*, with even evidence for more ways out of the periplasm, putatively multidrug‐efflux systems (Henríquez et al. 2019), no transporter taking over PvdE's function is known to be able to transport ferribactin into the periplasm. PvdE is a homolog of MacB, a protein family known to transport toxins and siderophores in *Pseudomonas* (Greene et al. 2018), so we were looking for other MacB homologs in *P. donghuensis* R32's genome, but without any hits. Since the synthesis can vary among different *Pseudomonas* strains, the presence of an unknown transporter, or even spontaneous diffusion, cannot be excluded. Interestingly, another study on *P. aeruginosa pvdE* mutants resulted in reduced, but still visible fluorescence (Tsuda et al. 1995) on agar plates. After assessing the typical absorbance of the pyoverdine fluorophore at 400 nm, they could establish that the remaining fluorescence visible on the agar plates came from other molecules. This possibility could be refuted in our case, since the absorbance of the *pvdE* mutants also shifted after acidification of the supernatant, like the wild‐type (data not shown).

We had previously also knocked out PvdQ in *P. donghuensis* R32, an enzyme that acts on ferribactin in the periplasm. It was reported that mutating this gene was preventing the synthesis of mature, fluorescent and functional pyoverdine (Hannauer et al. 2012; Wurst et al. 2014), yet fluorescence measurements of our R32 *pvdQ* mutants showed fluorescence levels comparable to the wild‐type levels, therefore we decided to continue our work with the *pvdE* mutants, under the assumption that a sevenfold reduction in pyoverdine levels (Figure 1A) would be enough to assess the relevance of pyoverdine in the strain's anti‐*Phytophthora* activity. The growth curves in LB supplemented with EDTA confirmed that our mutants had a significant phenotype, since the single and double *pvdE* mutants of both strains had almost suppressed growth (Figure S1). It seems that EDTA chelated the entire pool of previously freely available iron in LB. Only pyoverdine, which has an uncommonly high affinity for iron among siderophores (Dell'Anno et al. 2022; Meyer and Hornsperger 1978; Philson and Llinás 1982), could access this EDTA‐bound iron pool, thereby allowing the strains to grow normally. Other siderophores that might be present in the *pvdE* mutants apparently have lower affinities for iron than EDTA, and therefore do not allow the strains to acquire the necessary iron to grow normally. We hypothesize that, in contrast to LB, KB and V8 had a pool of already chelated iron before the addition of EDTA that pyoverdine mutants could still access, leading to reduced but significant growth in these two media.

### 4.1 The Discrepancy Between Siderophore Detection With CAS and With Fluorescence Suggests the Presence of Additional Siderophore(s)

When measuring the pyoverdine‐specific fluorescence levels in KB liquid culture, we could clearly observe higher amounts of pyoverdine in R32 than in R47 (Figures 1A and 2A). In contrast, in the fluorescence pictures we could see a more pronounced fluorescence intensity in R47 than in R32 (Figures 1B and 2B). Under broad‐spectrum UV, other molecules would fluoresce, which explains the observed discrepancy.

Interestingly, the reduced levels of pyoverdine in all four *pvdE* mutants were visible on the fluorescent plates but not reflected in a significantly reduced halo size in the CAS assay (Figure 1B), which is a clear indicator that both R32 and R47 might secrete one or multiple, yet unknown siderophores, in addition to pyoverdine. For *P. donghuensis* R32, we could indeed find a biosynthesis gene cluster for a siderophore which was not detected in our previous analysis of the strain's genome (De Vrieze et al. 2020): 7‐hydroxytropolone (7HT). This small molecule is known to be a siderophore, which is not fluorescent (Jiang et al. 2016) but would be visible on CAS, which would align well with the findings described above (Figure 1B). 7HT was reported to be the driver of antimicrobial activity of other *P. donghuensis* strains (Agaras et al. 2018; Munier‐Lépinay et al. 2025; Muzio et al. 2020). Another recent study even showed that 7HT was important for anti‐*Phytophthora* activity, as the double KO mutant of HCN and 7HT of their strain had reduced ability to inhibit the oomycete (Moffat et al. 2024). This strongly suggests that *P. donghuensis* R32 also uses 7HT to inhibit *P. infestans*, and that this siderophore might take over the iron‐scavenging activity in the *pvdE* mutants. The redundant iron competition mechanism of both pyoverdine and 7HT could also explain why the lack of pyoverdine did not impair our strains' ability to inhibit *P. infestans*, against our prior expectations. We know that 7HT and pyoverdine are linked in many ways in different *P. donghuensis* strains. It was suggested that they have complementary roles: 7HT being a small, low‐cost siderophore but with modest iron‐affinity, and pyoverdine being the contrary, the interplay would allow the cell to balance iron‐acquisition needs and metabolic costs (Jiang et al. 2016). This is underlined by the fact that both biosynthesis pathways are regulated by the GacS/GacA cascade, while only pyoverdine is regulated by the ferric uptake regulator, which allows strong upregulation when iron is needed in the cell (Jafra et al. 2023). Both are also affected by the global stress sigma factor SigW; it lowers 7HT production while permitting pyoverdine synthesis under iron stress conditions in strain HYS (Teng et al. 2023). Although it was reported that knocking out 7HT production in this strain did not affect pyoverdine synthesis (Chen et al. 2018), the contrary was observed for another strain (P482), where it seemed that the synthesis of one siderophore was a prerequisite for the synthesis of the other (Jafra et al. 2023). To know more about their complementary or redundant effect on the anti‐*Phytophthora* activity of *P. donghuensis* R32, a double KO mutant of both siderophores would be needed.

### 4.2 The Lack of Pyoverdine Only Affected the Performance of *P. chlororaphis* R47 in Leaf Disc Assays

In the case of *P. chlororaphis* R47, the impact of pyoverdine on mycelium growth inhibition was difficult to assess, since the high inhibitory activity was maintained for all mutants, across all iron supplementation levels (Figure 3). This was likely due to the phenazines produced by *P. chlororaphis* R47 (De Vrieze et al. 2020), which are known to be efficient biocontrol‐associated molecules (Biessy and Filion 2018) and have been previously demonstrated to restrict the mycelial growth of *P. infestans* (Morrison et al. 2017). Therefore, a putative impact of pyoverdine might have been masked by the presence of phenazines in this strain.

Evaluating pyoverdine's contribution to the inhibition of spore‐related developmental stages of *P. infestans* by *P. chlororaphis* R47 was just as difficult, this time for the opposite reason—a very weak or even absent activity in the wild‐type despite using higher cell densities than for *P. donghuensis* R32 in these experiments (Figures 4, 5, 6). This lack of activity differs from earlier experiments (Anand et al. 2020), but was stable across eight biological replicates and all three tested pathogen genotypes. Nevertheless, although we could not observe any significant decrease in germination percentage in *P. chlororaphis* R47‐exposed sporangia, the length of the germ tube was significantly decreased in contact with *P. chlororaphis* R47 wild‐type. Loss of pyoverdine did not affect this activity, in contrast to the loss of HCN, which increased it in both single and double mutants (Figure 6F), suggesting the upregulation of another trait involved in the inhibition of germ tube elongation.

In contrast, the results obtained in planta for *P. chlororaphis* R47 and its mutants clearly showed an impact of both pyoverdine and HCN on disease control: while the wild‐type protected the leaf discs successfully from lesion and mycelium formation, all three mutants lost their protective activity (Figure 7), suggesting that both traits are important determinants of in planta protection. Rather than only partially losing their protection, the single mutants were not more efficient than the double mutants. This could be explained by a regulatory interdependence of pyoverdine and HCN. While loss of HCN is expected to lead to higher pyoverdine levels (Anand et al. 2023), which was observed only in *P. donghuensis* R32, but not in *P. chlororaphis* R47 in the present study (Figure 1A), we do not know whether loss of pyoverdine would influence HCN emission on leaf discs. Since both molecules have the ability to bind iron and since HCN emission is higher in iron‐replete conditions (Askeland and Morrison 1983; Knowles and Bunch 1986), lower iron availability due to lack of pyoverdine might have had an impact on HCN emission. Alternatively, both molecules might have a redundant, iron‐scavenging or iron‐sensing function on leaf discs, which would also explain the lack of difference in efficacy between the two single and the double mutant. This lack of protection of all three mutants also suggests that phenazines may not be as important to inhibit mycelium development in planta as in vitro, a tendency that was already observed previously (Morrison et al. 2017). More importantly, we observed a loss of biocontrol activity in the pyoverdine mutants in this experiment, highlighting for the first time a certain relevance of this siderophore for the control of potato late blight. The fact that such an effect was not observed in *P. donghuensis* R32 might be due to the presence of the other siderophore mentioned above.

For *P. donghuensis* R32, the results obtained present themselves quite differently, which underlines that the two strains, although both *Pseudomonas* displaying antagonistic activity against *P. infestans*, likely employ different modes of action. In the mycelium growth inhibition under low iron conditions (no added FeCl_3_), the strongly reduced amount of pyoverdine had no impact on the level of inhibition in both the single and double mutants (Figure 3). This suggests that although iron competition plays a role in the interaction, which was evident from the decreased inhibition observed upon increasing iron supplementation, pyoverdine itself is not relevant for mycelial growth inhibition. More so, if pyoverdine was important for inhibiting this stage of the pathogen, we would expect a cumulative effect of the loss HCN (which resulted in reduced activity in the ∆*hcn* mutant) and pyoverdine, but we rather measured a significantly increased activity in the double mutant compared with the ∆*hcn* mutant, independently of iron supplementation. This increase in inhibition could be explained by the upregulation of one or multiple other biocontrol traits, such as 7HT, as suggested above.

When looking at the plates supplemented with iron (Figures 2B and 3B), we saw that *P. donghuensis* R32 and its mutants secreted a reddish molecule. Since we externally added high quantities of FeCl_3_ into the medium, but did not detect a toxic effect on bacterial growth (Figure S3), we could imagine that the red molecule is either oxidized, ferric iron, which was solubilized by the bacteria and secreted again, or even a pigment preventing iron‐mediated oxidative stress, as the prodigiosin known to be produced by *S. marcesens* and endorsing this oxidative stress alleviating function (Ma et al. 2025). Colonies were also smaller and thicker, a phenomenon of changed morphology that was also observed in *P. aeruginosa* and *Burkholderia cenocepacia* (Berlutti et al. 2005), where iron addition triggered increased biofilm formation.

In zoospore release and zoospore germination, the strong decrease of pyoverdine did not reduce the activity in *P. donghuensis* R32, and neither did the loss of HCN (Figures 4 and 5). However, the loss of both biocontrol traits did result in a strong reduction or even complete loss of inhibition activity, suggesting that both traits are equally efficient and sufficient when present alone to inhibit zoospores, but other traits play no or only very minor roles in this process. Another hypothesis that could explain our findings is that in the case of the pyoverdine single mutant, HCN is acidifying the growth medium, or even acting as a weak organic chelator (Rijavec and Lapanje 2016), so that the bacteria can take up iron more easily (Dumas et al. 2013; Loper and Henkels 1999), also with strongly reduced pyoverdine levels. This would also explain the need to produce more pyoverdine in the single HCN mutant (Figure 1A). In the double mutant, both pyoverdine‐mediated chelation and iron‐mediated acidification would be missing, which would explain the stronger loss of inhibition observed in zoospore release and germination assays.

We expected results from leaf disc assays to reflect those obtained from in vitro zoospore germination assays, but it was surprisingly not the case: both single and double mutants of *pvdE* conferred higher protection than *P. donghuensis* R32 wild‐type (Figure 7), suggesting once more the involvement of another siderophore, putatively 7HT (Chen et al. 2018; Jafra et al. 2023).

### 4.3 The Putative Functions of Pyoverdine Beyond Competition for Iron

The importance of pyoverdine as a mediator of competition for iron between *Pseudomonas* and *P. infestans* was minor in most of our findings, with the exception of the protective ability of *P. chlororaphis* R47 in leaf disc assays. This was surprising, since pyoverdine is known to have an especially high affinity for iron and is thought to be the main mechanism of iron acquisition in fluorescent *Pseudomonas* (Poole 2003). It is also being reported as the main driver of antagonism in various studies (Duijff et al. 1993; Gu et al. 2020; Liu et al. 2021; Ran et al. 2005; Sass et al. 2018; Sharifi et al. 2010). Yet, it is important to keep in mind the context of these studies, such as the targeted pathogen or experimental setup. Liu and colleagues, for example, observed pyoverdine to be the main driver of antibacterial activity in their *Pseudomonas* strain in iron‐starved conditions, but under nonstarving conditions, pyoverdine was not relevant for the activity anymore (Liu et al. 2021). In the studies by Ran et al. and Gu et al. although pyoverdine was assumed to be the main driver of the competition for iron responsible for the observed antagonism, they tested in both cases mutants that were deficient in two siderophores, making it difficult to conclude about the specific role of pyoverdine (Gu et al. 2020; Ran et al. 2005). This issue was also reported by Maldonado‐González et al. (2015), where they could not rule out the presence of an additional siderophore, which might have supplanted pyoverdine's function in the deficient mutant, and thus maintained its biocontrol activity. Other studies report that although pyoverdine was produced by the model strain analyzed, it was not the main mechanism of action in the strain's biocontrol activity (Djavaheri et al. 2012; Maldonado‐González et al. 2015; Ongena et al. 2002). This does not mean that pyoverdine, and in general siderophores, are not important biocontrol traits, but suggests that their role is strongly dependent on exogenous iron levels, other metabolites produced by the strain, the host plants, and the target pathogens.

Along the same lines, the limited role of pyoverdine in the anti‐*Phytophthora* activity of our two *Pseudomonas* strains does not mean that pyoverdine is not important for other functions in the strains. In multiple biological assays, we observed an increase in biocontrol activity when pyoverdine, or both pyoverdine and HCN, were knocked out. Similar to our previous findings that HCN is not only a toxin, but also a signaling molecule (Anand et al. 2023), there is evidence pointing to an indirect impact of the loss of pyoverdine on the R32's overall biocontrol activity, potentially resulting from the upregulation of other biocontrol traits triggered by reduced iron concentrations in the bacteria.

Iron is an important determinant of the whole regulatory machinery in bacteria (Hantke 2001). Pyoverdine might not only be used to acquire iron but might also contribute to iron homeostasis in the bacterial cell (Schalk and Guillon 2013). This implies that pyoverdine would be indirectly involved in the regulatory network of *Pseudomonas* strains. This hypothesis is supported by the fact that pyoverdine has an important role in biofilm formation and swarming (Kang et al. 2018; Kang and Kirienko 2017; Matilla et al. 2007). Matilla et al. even proposed that pyoverdine might be used in a kind of quorum‐sensing mechanism in which iron would be the signal molecule and pyoverdine the sensor (Matilla et al. 2007). In any case, the repeated observations that the lack of pyoverdine leads to higher antagonistic activity suggest that changes in iron sensing and homeostasis might be regulating biocontrol activity in antagonistic *Pseudomonas*.

The differing activities of our two strains and their respective mutants on the different developmental stages of *P. infestans* in vitro and in planta show that biocontrol is a complex phenomenon where the roles of specific molecules can be highly dependent on the producing strain, on the target pathogen, and on the conditions of the interaction (e.g., taking place in laboratory media or on plant material). Many studies show that indeed, there is a tripartite interaction taking place between plants, pathogens, and the surrounding microorganisms (Alfiky and Weisskopf 2021; Brotman et al. 2012; Hadizadeh et al. 2024; Marra et al. 2006), and that their interaction might be different than in a bipartite setup, since each interacting partner can emit molecules that could trigger gene expression changes in the others (Adeniji et al. 2020). Moreover, it is important to keep in mind that the results obtained on leaf discs, though in planta, may not be representative of what would happen on whole plants, or even in field conditions. Understanding the true role of pyoverdine in whole plant and field assays would therefore be an important avenue to explore in future studies, as well as elucidating the mechanisms leading to increased biocontrol activity in strains deprived of pyoverdine. Still, our findings can help improve concrete biocontrol strategies. Knowing mechanisms of action and biocontrol traits in specific conditions is very important and can help decide which traits might be interesting to boost in a formulated product. Our findings indicate that pyoverdine might not be the best candidate in this specific case to do so.

## 5 Conclusions

To assess the role of pyoverdine in the antagonistic activity of two potato‐associated *Pseudomonas* strains against *P. infestans*, we created pyoverdine mutants in different genetic backgrounds and evaluated their antagonistic effects on multiple developmental stages of the oomycete. We found little to no evidence of a direct contribution of pyoverdine to antagonistic activity. Instead, we found that the contribution of pyoverdine strongly depended on the *Pseudomonas* strain, and that its lack often resulted in higher antagonistic activity. This finding is unexpected, given pyoverdine's high affinity for iron and given the fact that *P. infestans* relies on iron supply for its growth and development (Cuppett and Lilly 1973). Iron is also relevant to the host plant potato, to defend itself against the oomycete upon infection (García Mata et al. 2001). All this, and the discovery that our strain *P. donghuensis* R32 additionally produces 7HT, shows the importance of iron and its acquisition for the virulence of *P. infestans* and for the protective activity of biocontrol strains. Further studies are needed to identify the mechanisms underlying the enhanced biocontrol activity observed in pyoverdine‐deficient mutants and to unravel how iron homeostasis regulates biocontrol‐relevant traits in plant‐associated *Pseudomonas*.

## Author Contributions

**Livia Jerjen:** conceptualization, investigation, writing – original draft, methodology, visualization, formal analysis, data curation. **Floriane L'Haridon:** methodology, investigation, writing – review and editing, formal analysis, supervision, resources. **Laure Weisskopf:** conceptualization, funding acquisition, writing – review and editing, validation, project administration, supervision, resources.

## Ethics Statement

The authors have nothing to report.

## Consent

The authors have nothing to report.

## Conflicts of Interest

The authors declare no conflicts of interest.

## Supporting information


**Figure S1:** Growth curves of *P. donghuensis* R32 and *P. chlororaphis* R47 genotypes under varying iron conditions. Growth of R32 and R47 and their respective mutants was monitored during 48 h in different liquid growth media. The panels to the left show growth in LB, the middle panels growth in KB and the right panels growth in filtered V8. The upper panels show growth in unmodified media, the middle panels show growth in media supplemented with 27 mg·L^−1^ FeCl_3_, and the lower panels show growth in media supplemented with 1 mM of the iron chelator EDTA.

**Figure S2:** Pyoverdine production monitored over time in varying media and temperatures. Pyoverdine production of both R32 and R47 and their mutants was monitored in KB (upper panels) and filtered V8 (lower panels), at 21°C (left panels) and 28°C (right panels). Pyoverdine was measured by fluorescence at 405 nm excitation and 460 nm emission wavelengths. The empty medium fluorescence was subtracted from the strains' fluorescent readings.

**Figure S3:** Assessment of growth during dual assays on V8 medium with different iron concentrations. Bacterial colonies were collected after 12 days of dual assay and CFUs were counted. The control condition (0 mg·L^−1^ added FeCl_3_) was compared to the 3 different FeCl_3_ concentrations (1 mg·L^−1^, 12 mg·L^−1^, 27 mg·L^−1^). Statistical analysis was performed using a t‐test comparing every condition to the control condition. No significant changes could be detected.

**Figure S4:** *P. infestans* zoospore release in presence of *P. donghuensis* R32 and *P. chlororaphis* R47. Percentages of empty sporangia exposed to bacteria. Bars represent the mean of three biological replicates with three technical replicates each. Statistical analysis was performed using a Kruskal–Wallis multiple comparisons test, followed by Dunn's test. Zoospores were exposed to: A: *P. infestans* strain Rec01 sporangia exposed to bacteria at OD_600_ = 0.25, B: *P. infestans* strain Rec01 sporangia exposed to bacteria at OD_600_ = 0.5, C: *P. infestans* strain 44 sporangia exposed to bacteria at OD_600_ = 0.25, D: *P. infestans* strain 44 sporangia exposed to bacteria at OD_600_ = 0.5.

**Figure S5:** *P. infestans* zoospore germination in presence of *P. donghuensis* R32 and *P. chlororaphis* R47. Percentages of germinated zoospores in treatments exposed or not exposed to the different strains are shown. Bars represent the mean of three biological replicates with three technical replicates each. Statistical analysis was performed using a Kruskal–Wallis multiple comparisons test, followed by Dunn's test. Zoospores were exposed to: A: *P. infestans* strain Rec01 zoospores exposed to bacteria at OD_600_ = 0.25, B: *P. infestans* strain Rec01 zoospores exposed to bacteria at OD_600_ = 0.5, C: *P. infestans* strain 44 zoospores exposed to bacteria at OD_600_ = 0.25, D: *P. infestans* strain 44 zoospores exposed to bacteria at OD_600_ = 0.5.

**Figure S6:** *P. infestans* sporangia germination in presence of *P. donghuensis* R32 and *P. chlororaphis* R47. Percentages of germinated sporangia in treatments exposed or not exposed to the different strains are shown. Bars represent the mean of three biological replicates with three technical replicates each. Statistical analysis was performed using a Kruskal–Wallis multiple comparisons test, followed by Dunn's test. Sporangia were exposed to: A: *P. infestans* strain GFP sporangia exposed to bacteria at OD_600_ = 0.1, B: *P. infestans* strain GFP sporangia exposed to bacteria at OD_600_ = 0.25, C: *P. infestans* strain Rec01 sporangia exposed to bacteria at OD_600_ = 0.1, D: *P. infestans* strain Rec01 sporangia exposed to bacteria at OD_600_ = 0.25, E: *P. infestans* strain 44 sporangia exposed to bacteria at OD_600_ = 0.1, F: *P. infestans* strain 44 sporangia exposed to bacteria at OD_600_ = 0.25.

**Figure S7:** Survival of *P. donghuensis* R32 and *P. chlororaphis* R47 genotypes on potato leaf discs. After 7 days of incubation on potato leaf discs with the same conditions used for the leaf disc assays, we recovered the bacteria on 3 single leaf discs per treatment, plated them and counted CFUs. No significant differences in survival ability were detected between the genotypes.

**Table S1:** List of strains and plasmids used for knocking out *pvdE* in *P. donghuensis* R32 and *P. chlororaphis* R47.

**Table S2:** List of primers used for knocking out *pvdE* in R32 and R47. Underlined sequences contain restriction sites necessary for truncating the target gene.

**Table S3:** Statistical analysis of results of pyoverdine measurements with FeCl_3_ supplementation (Figure 2). We performed a mixed‐effects model analysis with Geisser–Greenhouse correction followed by Tukey's test with two biological replicates with three technical replicates pooled together. In the first part of the table, the effect of the genotypes within the different iron concentrations is shown, whereas in the second part, the effect of the iron supplementation within the genotypes is shown. The adjusted p‐value is depicted on the left side of each cell, the significance on the right (∗ = *p* < 0.05, ∗∗ = *p* < 0.01, and ∗∗∗ = *p* < 0.001).

**Table S4:** Statistical analysis of results of mycelium growth inhibition with FeCl_3_ supplementation (Figure 3). We performed a mixed‐effects model analysis with Geisser–Greenhouse correction followed by Tukey's test with two biological replicates with four technical replicates pooled together. In the first part of the table, the effect of the genotypes within the different iron concentrations is shown, whereas in the second part, the effect of the iron supplementation within the genotypes is shown. The adjusted p‐value is depicted on the left side of each cell, the significance on the right (∗ = *p* < 0.05, ∗∗ = *p* < 0.01, and ∗∗∗ = *p* < 0.001).

## Data Availability

The data that support the findings of this study are available in the Supporting Information of this article.
