# Supplementary figures and images for: Pyoverdine Plays Only a Minor, Strain‐Specific Role in the Inhibition of Phytophthora infestans by Pseudomonas Strains
Source: Microbiologyopen. 2026 May 28;15(3):e70316. doi: 10.1002/mbo3.70316 (PMC13239315; doi:10.1002/mbo3.70316)

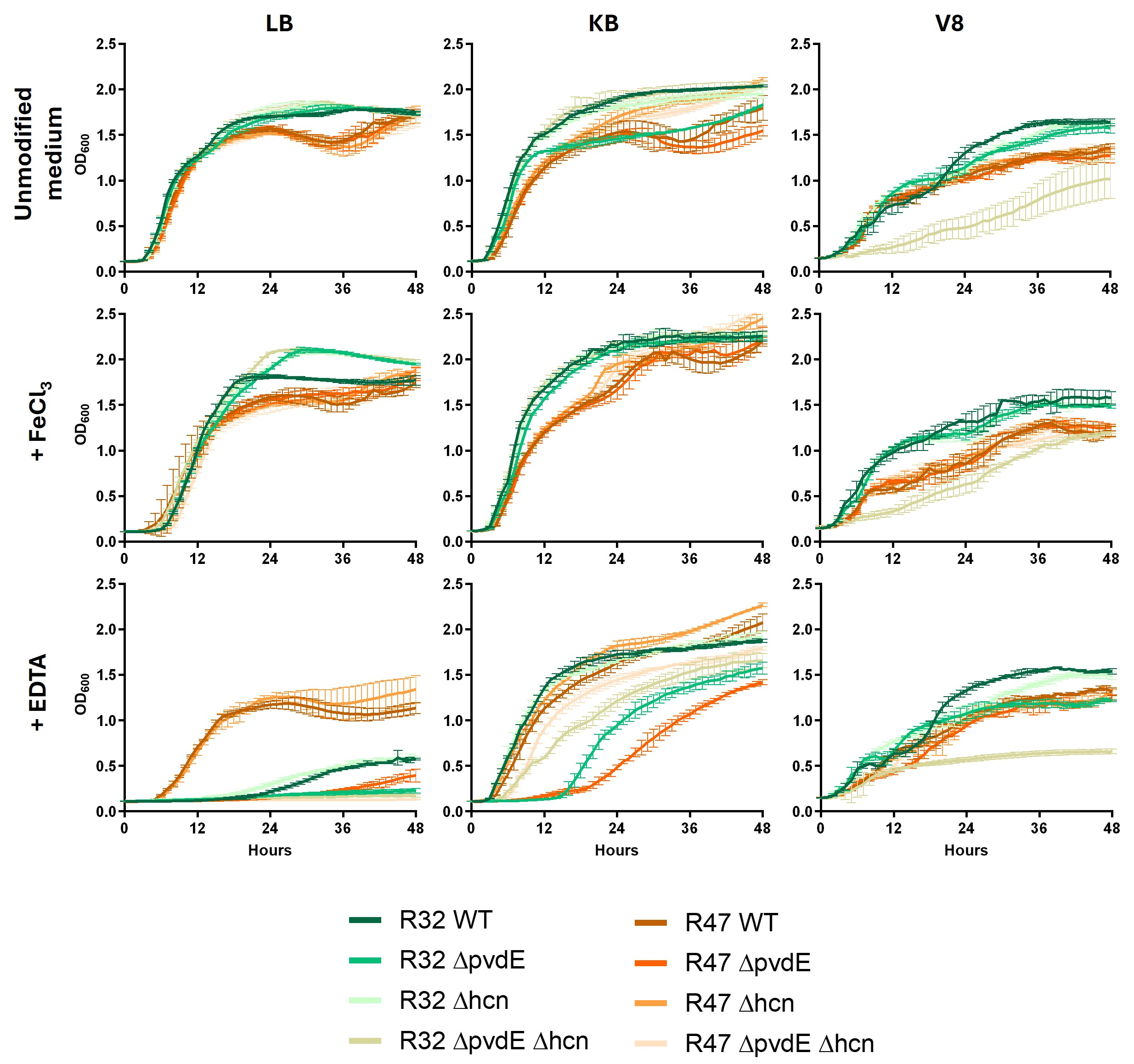

Supplement: Supplementary file 1 — Figure S1: Growth curves of P. donghuensis R32 and P. chlororaphis R47 genotypes under varying iron conditions. Growth of R32 and R47 and their respective mutants was monitored during 48 h in different liquid growth media. The panels to the left show growth in LB, the middle panels growth in KB and the right panels growth in filtered V8. The upper panels show growth in unmodified media, the middle panels show growth in media supplemented with 27 mg·L−1 FeCl3, and the lower panels show growth in media supplemented with 1 mM of the iron chelator EDTA. [file MBO3-15-e70316-s007.tif]

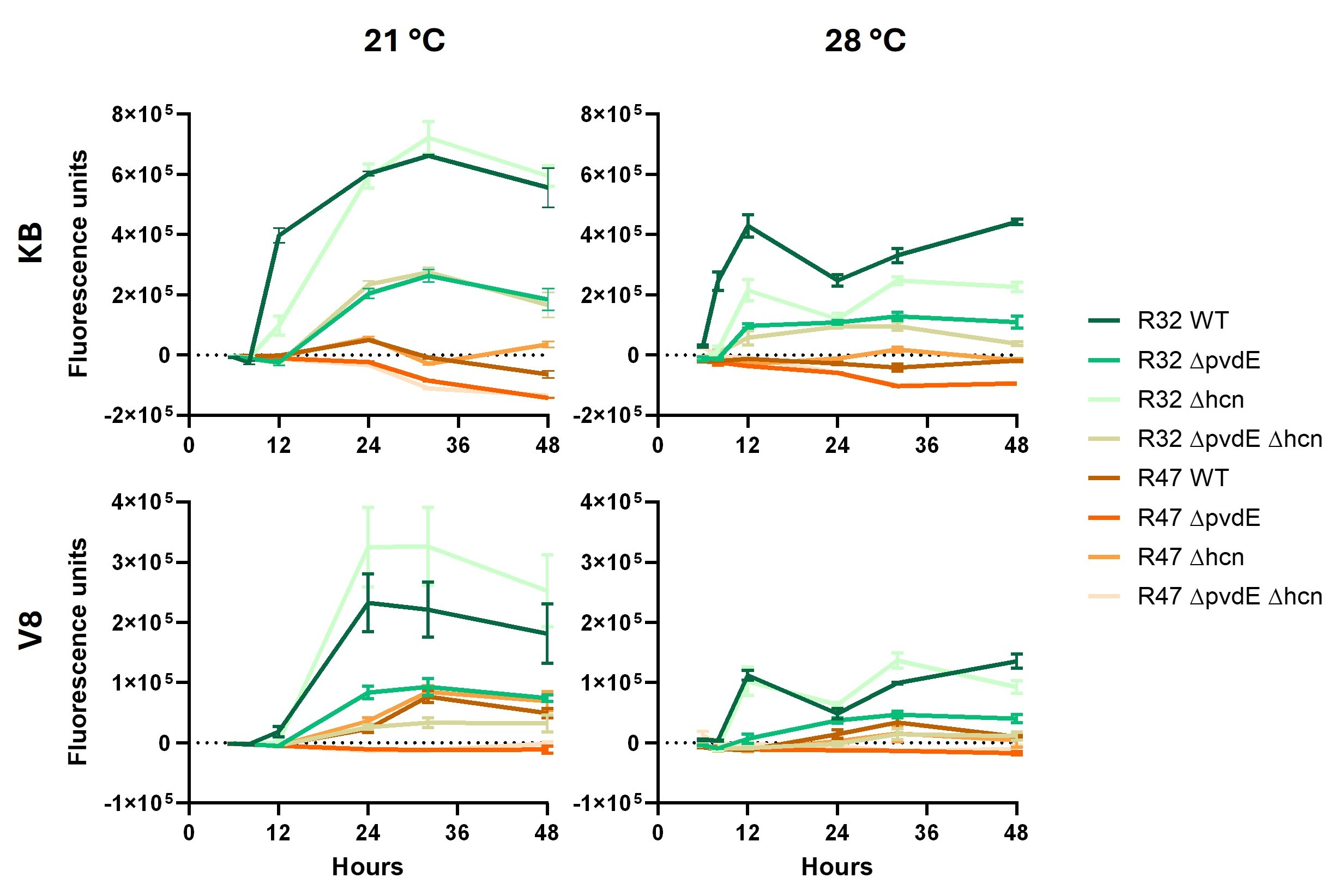

Supplement: Supplementary file 2 — Figure S2: Pyoverdine production monitored over time in varying media and temperatures. Pyoverdine production of both R32 and R47 and their mutants was monitored in KB (upper panels) and filtered V8 (lower panels), at 21°C (left panels) and 28°C (right panels). Pyoverdine was measured by fluorescence at 405 nm excitation and 460 nm emission wavelengths. The empty medium fluorescence was subtracted from the strains' fluorescent readings. [file MBO3-15-e70316-s008.tif]

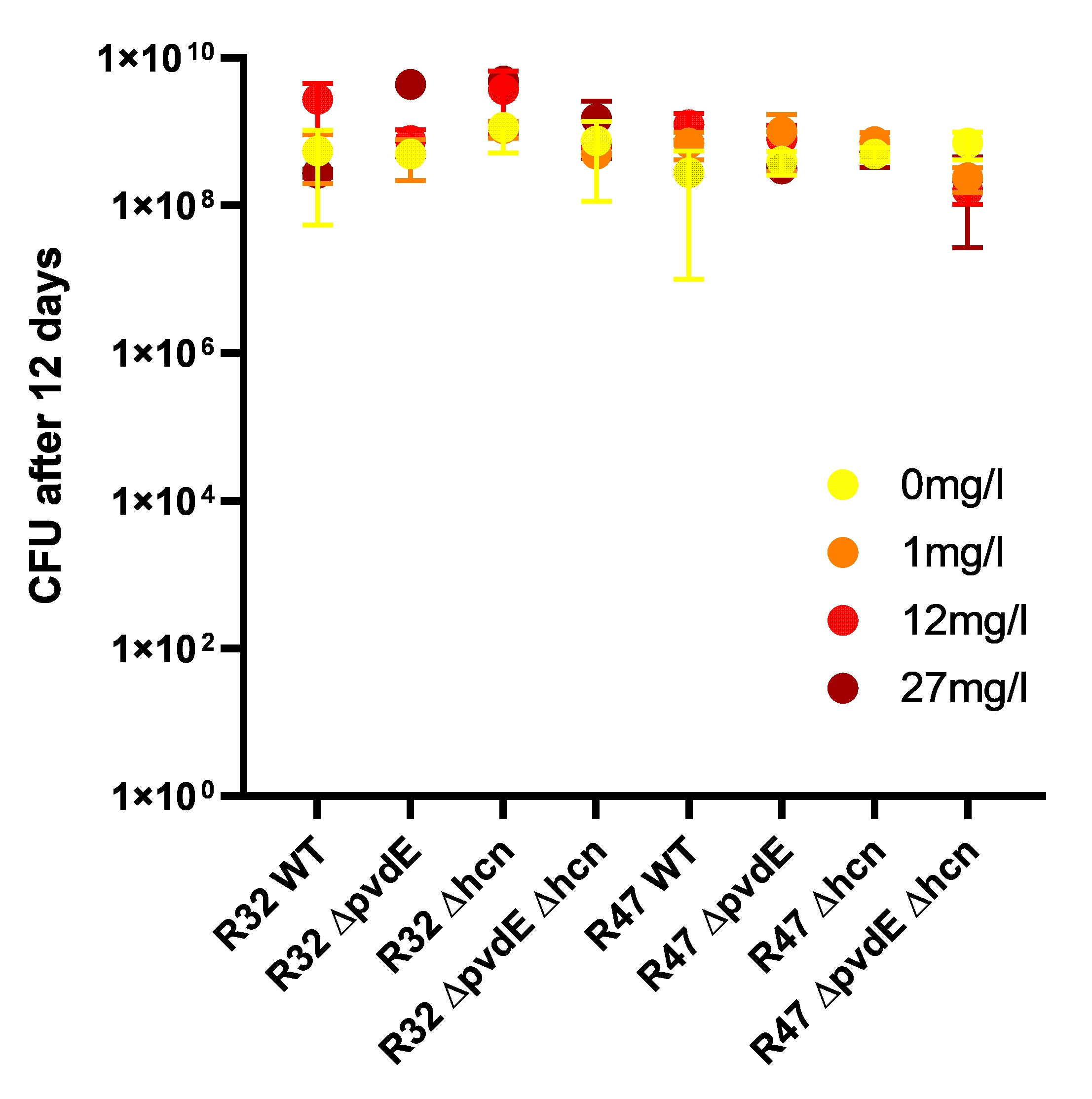

Supplement: Supplementary file 3 — Figure S3: Assessment of growth during dual assays on V8 medium with different iron concentrations. Bacterial colonies were collected after 12 days of dual assay and CFUs were counted. The control condition (0 mg·L−1 added FeCl3) was compared to the 3 different FeCl3 concentrations (1 mg·L−1, 12 mg·L−1, 27 mg·L−1). Statistical analysis was performed using a t‐test comparing every condition to the control condition. No significant changes could be detected. [file MBO3-15-e70316-s003.tif]

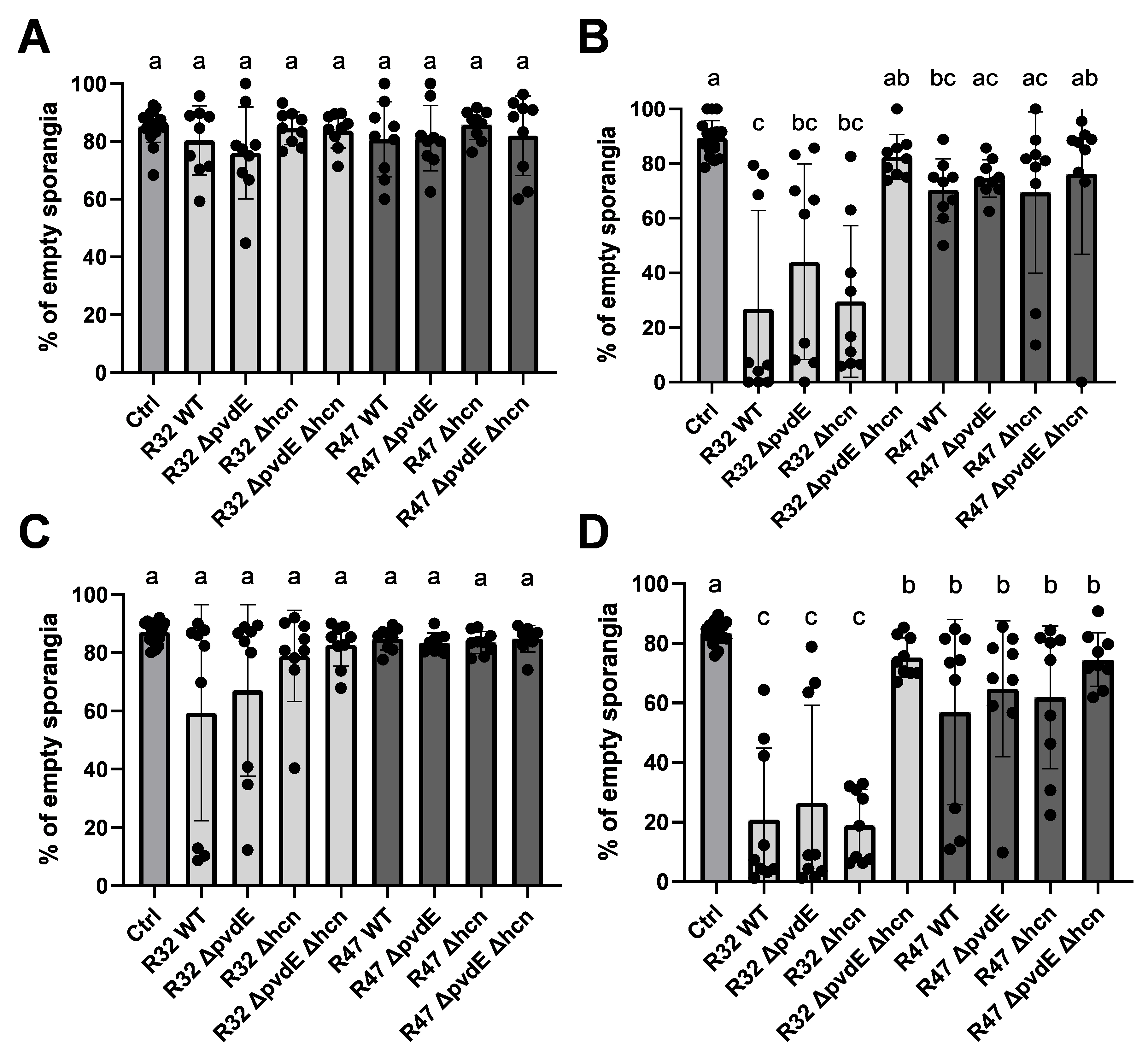

Supplement: Supplementary file 4 — Figure S4: P. infestans zoospore release in presence of P. donghuensis R32 and P. chlororaphis R47. Percentages of empty sporangia exposed to bacteria. Bars represent the mean of three biological replicates with three technical replicates each. Statistical analysis was performed using a Kruskal–Wallis multiple comparisons test, followed by Dunn's test. Zoospores were exposed to: A: P. infestans strain Rec01 sporangia exposed to bacteria at OD600 = 0.25, B: P. infestans strain Rec01 sporangia exposed to bacteria at OD600 = 0.5, C: P. infestans strain 44 sporangia exposed to bacteria at OD600 = 0.25, D: P. infestans strain 44 sporangia exposed to bacteria at OD600 = 0.5. [file MBO3-15-e70316-s005.tif]

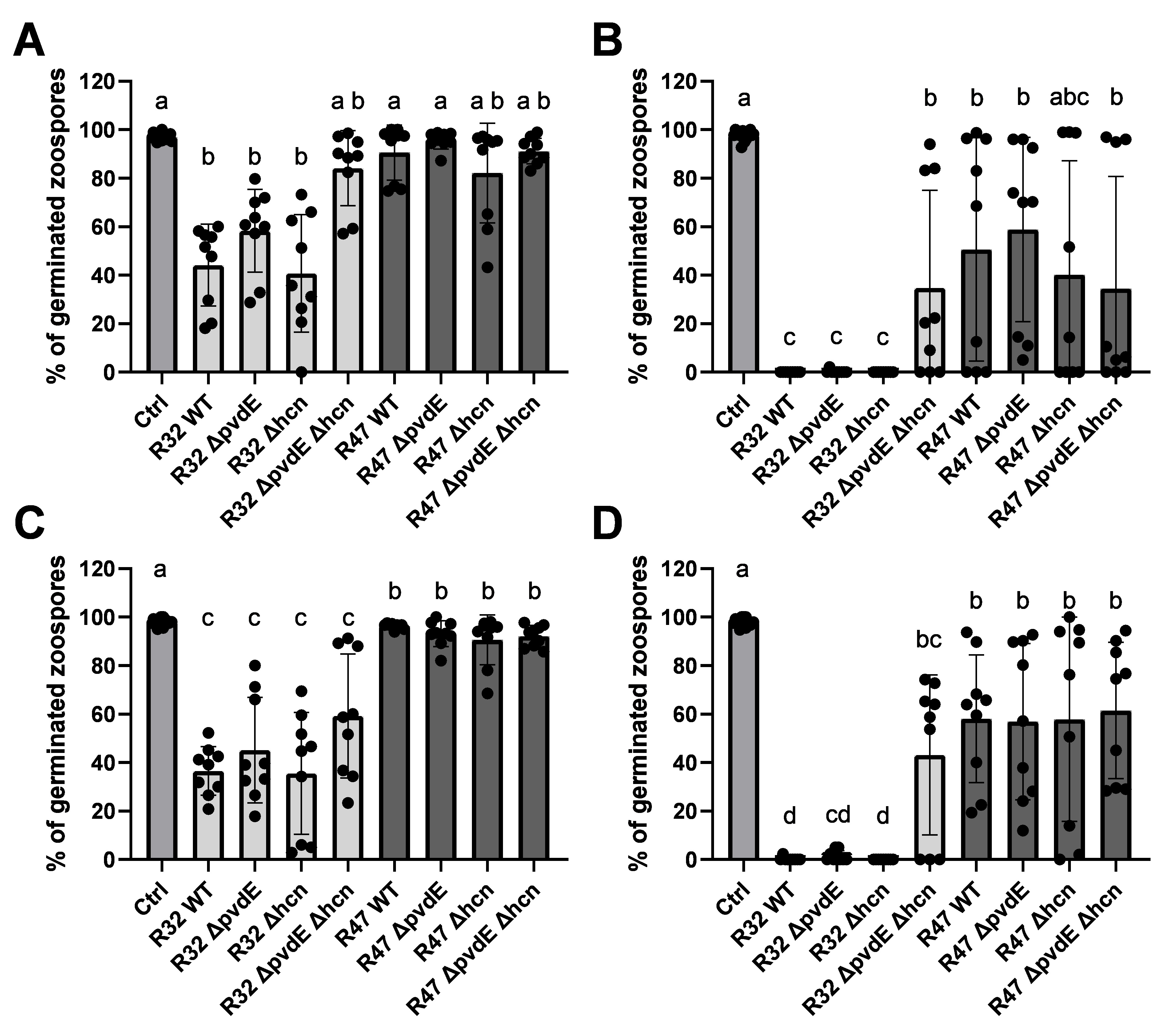

Supplement: Supplementary file 5 — Figure S5: P. infestans zoospore germination in presence of P. donghuensis R32 and P. chlororaphis R47. Percentages of germinated zoospores in treatments exposed or not exposed to the different strains are shown. Bars represent the mean of three biological replicates with three technical replicates each. Statistical analysis was performed using a Kruskal–Wallis multiple comparisons test, followed by Dunn's test. Zoospores were exposed to: A: P. infestans strain Rec01 zoospores exposed to bacteria at OD600 = 0.25, B: P. infestans strain Rec01 zoospores exposed to bacteria at OD600 = 0.5, C: P. infestans strain 44 zoospores exposed to bacteria at OD600 = 0.25, D: P. infestans strain 44 zoospores exposed to bacteria at OD600 = 0.5. [file MBO3-15-e70316-s006.tif]

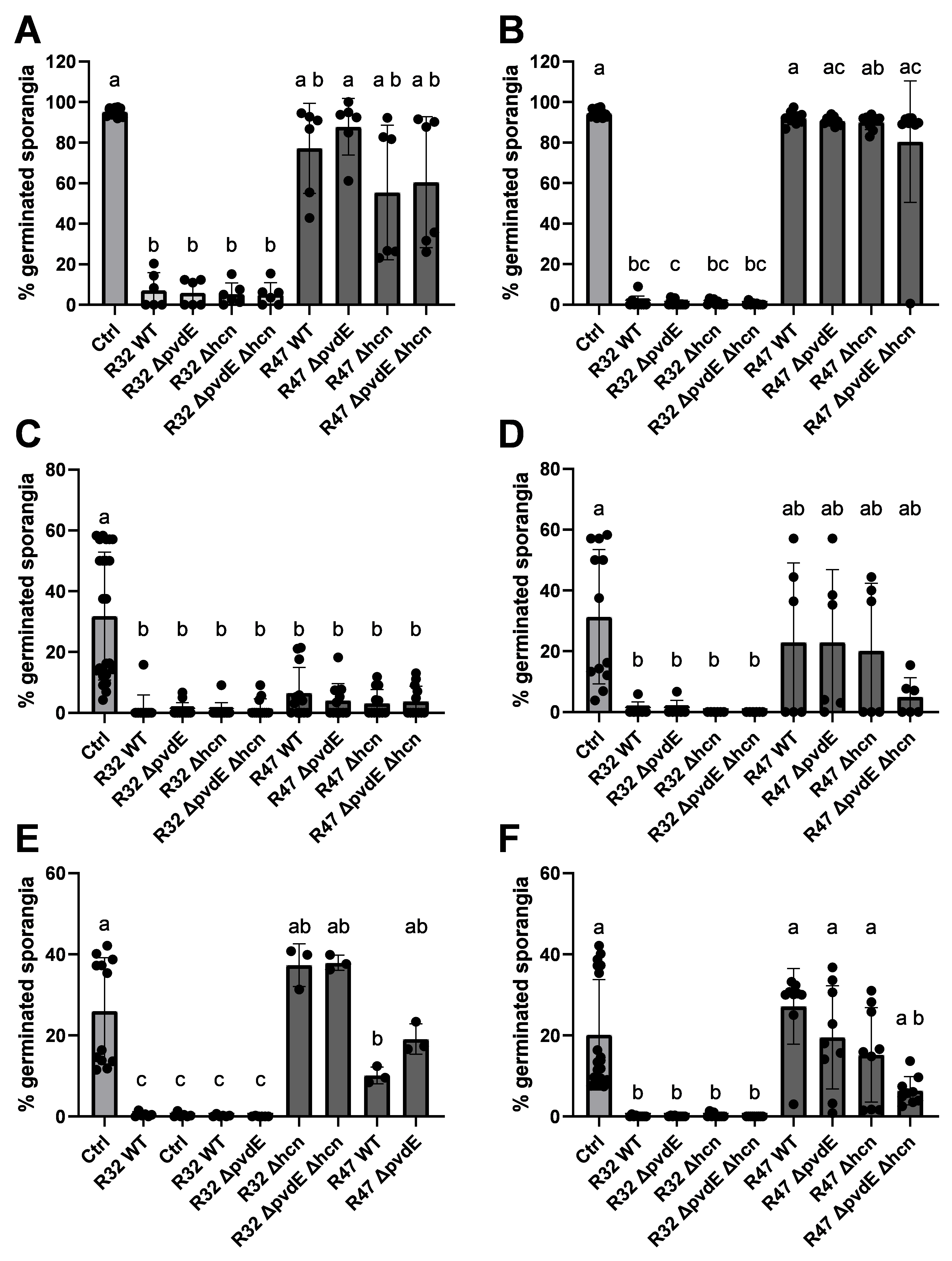

Supplement: Supplementary file 6 — Figure S6: P. infestans sporangia germination in presence of P. donghuensis R32 and P. chlororaphis R47. Percentages of germinated sporangia in treatments exposed or not exposed to the different strains are shown. Bars represent the mean of three biological replicates with three technical replicates each. Statistical analysis was performed using a Kruskal–Wallis multiple comparisons test, followed by Dunn's test. Sporangia were exposed to: A: P. infestans strain GFP sporangia exposed to bacteria at OD600 = 0.1, B: P. infestans strain GFP sporangia exposed to bacteria at OD600 = 0.25, C: P. infestans strain Rec01 sporangia exposed to bacteria at OD600 = 0.1, D: P. infestans strain Rec01 sporangia exposed to bacteria at OD600 = 0.25, E: P. infestans strain 44 sporangia exposed to bacteria at OD600 = 0.1, F: P. infestans strain 44 sporangia exposed to bacteria at OD600 = 0.25. [file MBO3-15-e70316-s004.tif]

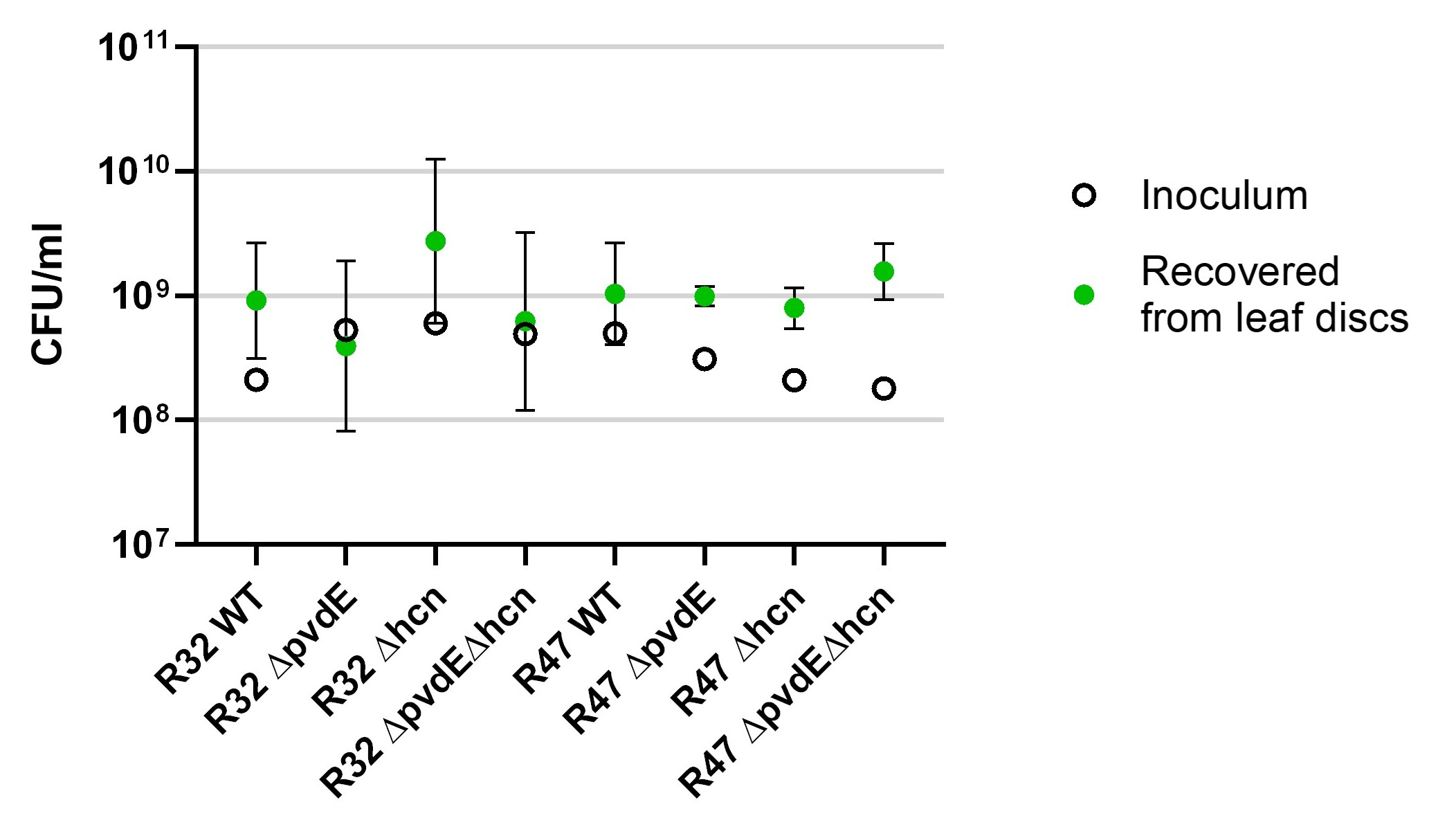

Supplement: Supplementary file 7 — Figure S7: Survival of P. donghuensis R32 and P. chlororaphis R47 genotypes on potato leaf discs. After 7 days of incubation on potato leaf discs with the same conditions used for the leaf disc assays, we recovered the bacteria on 3 single leaf discs per treatment, plated them and counted CFUs. No significant differences in survival ability were detected between the genotypes. [file MBO3-15-e70316-s001.tif]
