# Supplementary material for: Pyoverdine Plays Only a Minor, Strain‐Specific Role in the Inhibition of Phytophthora infestans by Pseudomonas Strains
Source: Microbiologyopen. 2026 May 28;15(3):e70316. doi: 10.1002/mbo3.70316 (PMC13239315; doi:10.1002/mbo3.70316)
Supplement: Supplementary file 8 — Table S1: List of strains and plasmids used for knocking out pvdE in P. donghuensis R32 and P. chlororaphis R47. [file MBO3-15-e70316-s002.docx]

**Table S1**

| **Strain/Plasmid** | **Relevant description** | **Reference** |
| --- | --- | --- |
| pEMG | Kanamycin^R^, *ori* R6K, *lacZ* α, I-SceI sites | Martínez-García et al., 2011 |
| pSW-2 | Gentamycin^R^, *ori* RK2, *I-sceI* I | Martínez-García et al., 2011 |
| pEMG::∆*pvdE*_R32WT | pEMG plasmid containing 1.2kb fragment 1-fragment 3 EcoRI-BamHI insert from R32WT for deleting *pvdE* | This work |
| pEMG::∆*pvdE*_R47WT | pEMG plasmid containing 1.4kb fragment 1-fragment 3 EcoRI-BamHI insert from R47WT for deleting *pvdE* | This work |
| R32 WT | Wild-type strain | Hunziker et al., 2015 |
| R47 WT | Wild-type strain | Hunziker et al., 2015 |
| R32 ∆*hcn* | ∆*hcn* in-frame deletion mutant of R32 wild-type | Anand et al., 2020 |
| R47 ∆*hcn* | ∆*hcn* in-frame deletion mutant of R47 wild-type | Anand et al., 2020 |
| R32 WT pEMG::∆*pvdE* | Wild-type strain with pEMG::del*pvdE*R32 plasmid, kmR | This work |
| R47 WT pEMG::∆*pvdE* | Wild-type strain with pEMG::del*pvdE*R47 plasmid, kmR | This work |
| R32 ∆*hcn* pEMG::∆*pvdE* | ∆*hcn* strain with pEMG::del*pvdE*R32 plasmid, kmR | This work |
| R47 ∆*hcn* pEMG::∆*pvdE* | ∆*hcn* strain with pEMG::del*pvdE*R47 plasmid, kmR | This work |
| R32 ∆*hcn* ∆*pvdE* | ∆*pvdE* in-frame deletion mutant of R32 ∆*hcn* | This work |
| R47 ∆*hcn* ∆*pvdE* | ∆*pvdE* in-frame deletion mutant of R47 ∆*hcn* | This work |
