# Supplementary material for: Pyoverdine Plays Only a Minor, Strain‐Specific Role in the Inhibition of Phytophthora infestans by Pseudomonas Strains
Source: Microbiologyopen. 2026 May 28;15(3):e70316. doi: 10.1002/mbo3.70316 (PMC13239315; doi:10.1002/mbo3.70316)
Supplement: Supplementary file 9 — Table S2: List of primers used for knocking out pvdE in R32 and R47. Underlined sequences contain restriction sites necessary for truncating the target gene. [file MBO3-15-e70316-s011.docx]

**Table S2**

| **Primer** | **Sequence; restriction sites (underlined)** | **Usage** |
| --- | --- | --- |
| R32 pvdE 1 | CGGGATCCGCCGACGAGATCAGCCAG | Verification of insertion in R32 |
| R32 pvdE4 | CGGAATTCTGGCCTGCTGGCCGAA | Verification of insertion in R32 |
| R47 pvdE1 | CGGGATCCCATGTAGCGCTCGTGA | Fragment 1 for R47 |
| R47 pvdE2 | GGGGTACCAGTGCAGGGCGTTGTT | Fragment 1 for R47 |
| R47 pvdE3 | GGGGTACCGCAAGACCATCATCGT | Fragment 2 for R47 |
| R47 pvdE4 | CGGAATTCGAACTCCTGCAGGGCC | Fragment 2 for R47 |
