# Supplementary material for: Pyoverdine Plays Only a Minor, Strain‐Specific Role in the Inhibition of Phytophthora infestans by Pseudomonas Strains
Source: Microbiologyopen. 2026 May 28;15(3):e70316. doi: 10.1002/mbo3.70316 (PMC13239315; doi:10.1002/mbo3.70316)
Supplement: Supplementary file 10 — Table S3: Statistical analysis of results of pyoverdine measurements with FeCl3 supplementation (Figure 2). We performed a mixed‐effects model analysis with Geisser–Greenhouse correction followed by Tukey's test with two biological replicates with three technical replicates pooled together. In the first part of the table, the effect of the genotypes within the different iron concentrations is shown, whereas in the second part, the effect of the iron supplementation within the genotypes is shown. The adjusted p‐value is depicted on the left side of each cell, the significance on the right (∗ = p < 0.05, ∗∗ = p < 0.01, and ∗∗∗ = p < 0.001). [file MBO3-15-e70316-s010.docx]

**Table S3**

| **R32** | **0 mg.L^-1^** | | **1 mg.L^-1^** | | **12 mg.L^-1^** | | **27 mg.L^-1^** | |
| --- | --- | --- | --- | --- | --- | --- | --- | --- |
| **WT vs. ∆*pvdE*** | <0.001 | *** | <0.001 | *** | 0.002 | ** | <0.001 | *** |
| **WT vs. ∆*hcn*** | <0.001 | *** | 0.206 | ns | 0.001 | ** | 0.051 | ns |
| **WT vs. ∆*pvdE* ∆*hcn*** | <0.001 | *** | <0.001 | *** | <0.001 | *** | <0.001 | *** |
| **∆*pvdE* vs. ∆*hcn*** | <0.001 | *** | 0.236 | ns | 0.06 | ns | <0.001 | *** |
| **∆*pvdE* vs. ∆*pvdE* ∆*hcn*** | 0.192 | ns | <0.001 | *** | 0.001 | ** | <0.001 | *** |
| **∆*hcn* vs. ∆*pvdE* ∆*hcn*** | <0.001 | *** | 0.002 | ** | <0.001 | *** | <0.001 | *** |
| **R47** | **0 mg.L^-1^** | | **1 mg.L^-1^** | | **12 mg.L^-1^** | | **27 mg.L^-1^** | |
| **WT vs. ∆*pvdE*** | <0.001 | *** | 0.021 | * | 0.563 | ns | 0.849 | ns |
| **WT vs. ∆*hcn*** | 0.998 | ns | 0.274 | ns | 0.686 | ns | 0.808 | ns |
| **WT vs. ∆*pvdE* ∆*hcn*** | <0.001 | *** | 0.073 | ns | 0.475 | ns | 0.512 | ns |
| **∆*pvdE* vs. ∆*hcn*** | 0.004 | ** | 0.033 | * | 0.985 | ns | 0.575 | ns |
| **∆*pvdE* vs. ∆*pvdE* ∆*hcn*** | 0.199 | ns | 0.699 | ns | >0.999 | ns | 0.386 | ns |
| **∆*hcn* vs. ∆*pvdE* ∆*hcn*** | 0.002 | ** | 0.049 | * | 0.973 | ns | 0.995 | ns |
|  |  |  |  |  |  |  |  |  |
| **R32** | **WT** | | **∆*pvdE*** | | **∆*hcn*** | | **∆*pvdE* ∆*hcn*** | |
| **0 mg.L^-1^ vs. 1 mg.L^-1^** | 0.016 | * | 0.026 | * | <0.001 | *** | 0.208 | ns |
| **0 mg.L^-1^ vs. 12 mg.L^-1^** | 0.004 | ** | 0.605 | ns | <0.001 | *** | 0.212 | ns |
| **0 mg.L^-1^ vs. 27 mg.L^-1^** | <0.001 | *** | 0.454 | ns | <0.001 | *** | 0.164 | ns |
| **1 mg.L^-1^ vs. 12 mg.L^-1^** | 0.363 | ns | <0.001 | *** | <0.001 | *** | 0.007 | ** |
| **1 mg.L^-1^ vs. 27 mg.L^-1^** | 0.03 | * | 0.001 | ** | 0.054 | ns | <0.001 | *** |
| **12 mg.L^-1^ vs. 27 mg.L^-1^** | 0.118 | ns | 0.929 | ns | <0.001 | *** | 0.241 | ns |
| **R47** | **WT** | | **∆*pvdE*** | | **∆*hcn*** | | **∆*pvdE* ∆*hcn*** | |
| **0 mg.L^-1^ vs. 1 mg.L^-1^** | <0.001 | *** | 0.056 | ns | 0.007 | ** | 0.851 | ns |
| **0 mg.L^-1^ vs. 12 mg.L^-1^** | <0.001 | *** | 0.048 | * | <0.001 | *** | 0.205 | ns |
| **0 mg.L^-1^ vs. 27 mg.L^-1^** | <0.001 | *** | 0.019 | * | <0.001 | *** | 0.108 | ns |
| **1 mg.L^-1^ vs. 12 mg.L^-1^** | <0.001 | *** | 0.033 | * | 0.007 | ** | 0.068 | ns |
| **1 mg.L^-1^ vs. 27 mg.L^-1^** | <0.001 | *** | 0.006 | ** | 0.004 | ** | 0.016 | * |
| **12 mg.L^-1^ vs. 27 mg.L^-1^** | 0.005 | ** | 0.561 | ns | 0.892 | ns | >0.999 | ns |
